# Stable cortical body maps before and after arm amputation

**DOI:** 10.1038/s41593-025-02037-7

**Published:** 2025-08-21

**Authors:** Hunter R. Schone, Roni O. Maimon-Mor, Mathew Kollamkulam, Malgorzata A. Szymanska, Craig Gerrand, Alexander Woollard, Norbert V. Kang, Chris I. Baker, Tamar R. Makin

**Affiliations:** 1 Institute of Cognitive Neuroscience, University College London, London, UK; 2 Laboratory of Brain & Cognition, National Institutes of Mental Health, National Institutes of Health, Bethesda, MD USA; 3 Rehab Neural Engineering Labs, University of Pittsburgh, Pittsburgh, PA USA; 4 Department of Physical Medicine and Rehabilitation, University of Pittsburgh, Pittsburgh, PA USA; 5 Department of Experimental Psychology, University College London, London, UK; 6 UCL Institute of Ophthalmology, University College London, London, UK; 7 Department of Experimental Psychology, University of Oxford, Oxford, UK; 8 MRC Cognition and Brain Sciences Unit, University of Cambridge, Cambridge, UK; 9 Department of Orthopaedic Oncology, Royal National Orthopaedic Hospital NHS Trust, Stanmore, UK; 10 Plastic Surgery Department, Royal Free Hospital NHS Trust, London, UK; 11 Wellcome Centre for Human Neuroimaging, UCL Institute of Neurology, London, UK

**Keywords:** Motor control, Sensorimotor processing, Cortex, Cognitive neuroscience

## Abstract

The adult brain’s capacity for cortical reorganization remains debated. Using longitudinal neuroimaging in three adults, followed before and up to 5 years after arm amputation, we compared cortical activity elicited by movement of the hand (before amputation) versus phantom hand (after amputation) and lips (before and after amputation). We observed stable cortical representations of both hand and lips in primary sensorimotor regions. By directly quantifying activity changes across amputation, we demonstrate that amputation does not trigger large-scale cortical reorganization.

## Abstract (web summary)

Longitudinal neuroimaging of participants with planned arm amputations shows that the cortical body map remains stable after amputation, with no evidence of hand or face reorganization, thus challenging long-standing theories of brain remapping after limb loss.

## Main

What happens to the brain’s map of the body when a part of the body is removed? Over the last five decades, this question has captivated neuroscientists and clinicians, driving research into the brain’s capacity to reorganize itself. Primary somatosensory cortex (S1), known for its highly detailed body map, has historically been the definitive region for studying cortical reorganization^1,2^. For example, foundational research in monkeys reported that, after an amputation or deafferentation, the affected region within the S1 body map suddenly responds to inputs from cortically neighboring body parts (for example, the face)^3,4^. Additional neuroimaging studies in human amputees supported the theory that amputation of an arm triggers large-scale cortical reorganization of the S1 body map^5–7^, with a dramatic redistribution of cortical resources, hijacking the deprived territory^1^.

Recent studies have challenged this view by harnessing human amputees’ reports of experiencing vivid sensations of the missing (phantom) limb. First, human neuroimaging studies demonstrated that voluntary movements of phantom fingers engage neural patterns resembling those of able-bodied individuals^8–10^. Second, phantom sensations are evoked by cortical^11^ or peripheral^12,13^ nerve stimulation, suggesting an intact neural representation of the amputated limb, despite its physical absence. Third, neuroimaging studies using both tactile stimulation and movement paradigms reported no changes in face or lip activity within the deprived cortex of adult amputee participants compared to able-bodied controls^14,15^ (although remapping has been observed in children)^16^.

**Fig. 1:**
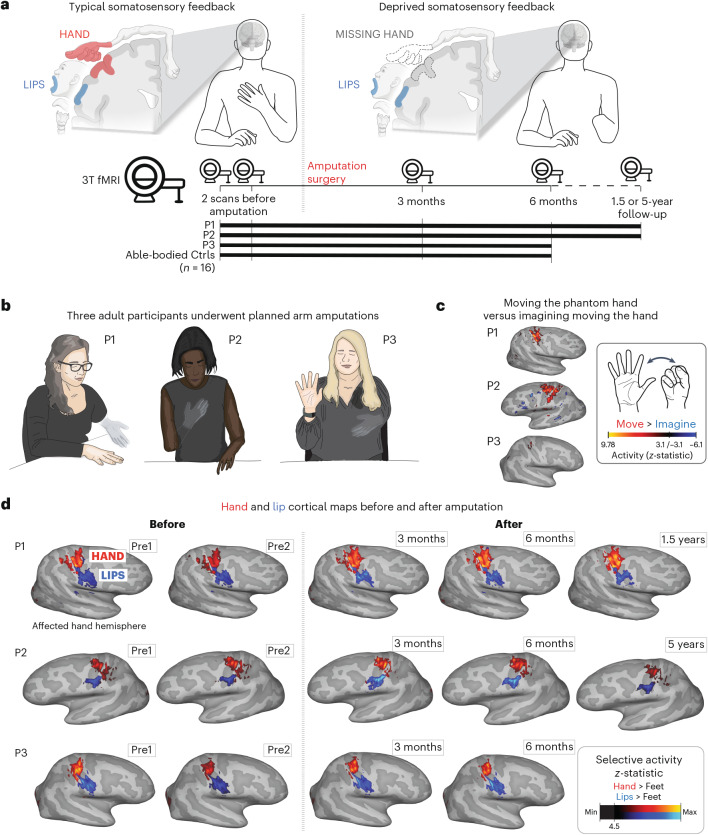
Longitudinal investigation of participants with planned arm amputations. **a**, Experimental timeline. Scans before and after amputation were conducted across 4–5 time points: twice before, and at 3 months, 6 months and 1.5 (P1)/5 years (P2) after amputation. **b**, Illustration depicting the three participants 6 months after amputation, including their subjective description of their phantom limb position. **c**, Phantom movements are not imaginary. Univariate activity (*z*-scored) contrast map displaying a participant’s attempts to open and close the phantom hand versus imagining movement, 6 months after amputation. **d**, Participant’s hand (red) and lip (blue) cortical activation maps (contrasted against feet movements) in the affected hand hemisphere across 4–5 sessions. All maps were minimally thresholded at 33% the maximum *z*-statistic and used a common color scale (the participant’s maximum *z*-statistic > 4.5). Participants agreed to have their image reproduced. Brain illustrations in **a** were created in BioRender.

This debate—whether or not amputation triggers large-scale reorganization—remains unresolved^17,18^, with some suggesting that the two views are not conceptually exclusive, that is, preservation and reorganization can coexist^5,19,20^. However, a fundamental issue with the evidence on both sides of this debate is a methodological reliance on cross-sectional designs (that is, comparisons between participants). While offering valuable proofs of concept, these studies cannot determine whether the maps of the phantom hand or face are truly preserved or changed relative to their pre-amputation state. To directly track the evolution of cortical representations before and after amputation, we implemented a longitudinal functional magnetic resonance imaging (MRI) approach to track the cortical representations of the hand and face (lips) in three adult participants up to 5 years after arm amputation (Supplementary Video 1), compared with able-bodied control participants (Ctrl) (Fig. 1a). Avoiding the confounding effects of cross-sectional designs^21^, we directly quantified the impact of arm amputation on S1 (re)organization.

**Extended Data Fig. 1:**
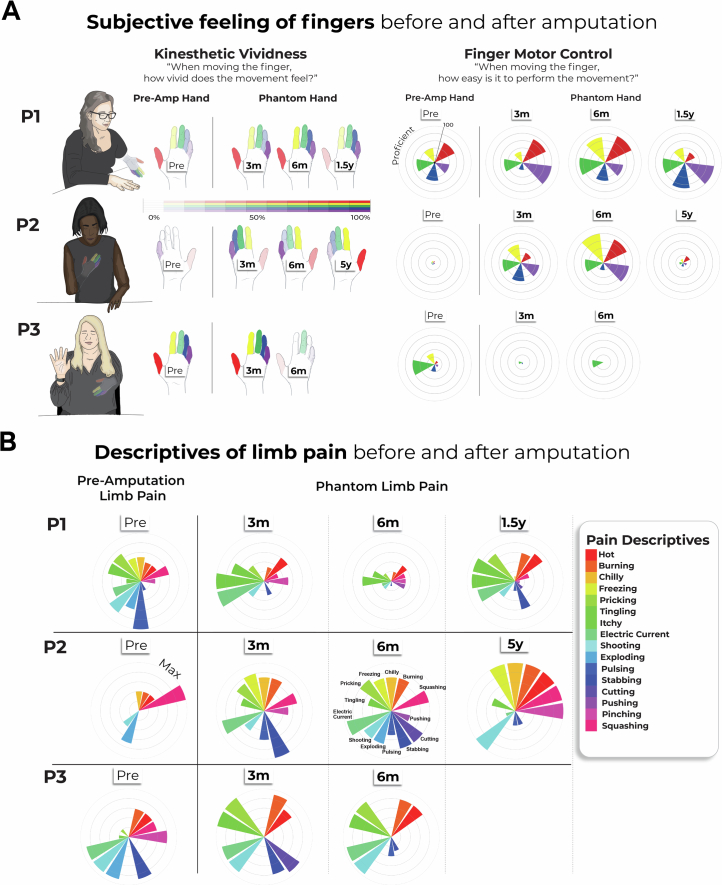
Longitudinal characterization of finger sensations and limb pain. (**a**) Affected hand sensations before and after amputation. Finger vividness and motor control for the phantom fingers, relative to the pre-amputated fingers. Kinesthetic vividness rated on a scale from 0 (no sensation) to 100 (as vivid as the unaffected hand) with color intensity indicating level. Movement difficulty rated from 100 (as easy as the unimpaired hand) to 0 (extremely difficult). Finger colors: red=D1, yellow=D2, green=D3, blue=D4, purple=D5 (palm excluded). (**b**) Before and after amputation, participants reported intensity values for each pain descriptive word, broadly categorized into sensations that are mechanical, temperature-related and other. For each word, participants were asked to describe the intensity between 0 (non-existing) to 100 (excruciating pain) as it relates to that particular word. A value of 100 (Max) is the largest radii on the polar plot. 3 M=3months post-amputation; 6 M=6months post-amputation. 1.5/5 yrs=1.5 or 5 years post-amputation.

We studied three adult participants (case studies P1, P2 and P3) undergoing arm amputation (demographics in Extended Data Table 1) across 4–5 time points, and 16 able-bodied Ctrls at four time points over 6 months (Fig. 1a). Before amputation, all participants could move all fingers to varying ranges (Extended Data Fig. 1 and Supplementary Video 2). After amputation, all participants reported vivid phantom limb sensations (Fig. 1b), including volitional phantom finger movement (Extended Data Table 1 and Extended Data Fig. 1). Motor control over the phantom hand was further confirmed by residual limb muscle contractions during phantom movements (Supplementary Video 2), and selective activation in primary sensorimotor cortex for attempted, but not imagined, phantom movements (Fig. 1c). The critical question is to what degree S1 phantom activity reflects the pre-existing hand.

**Fig. 2:**
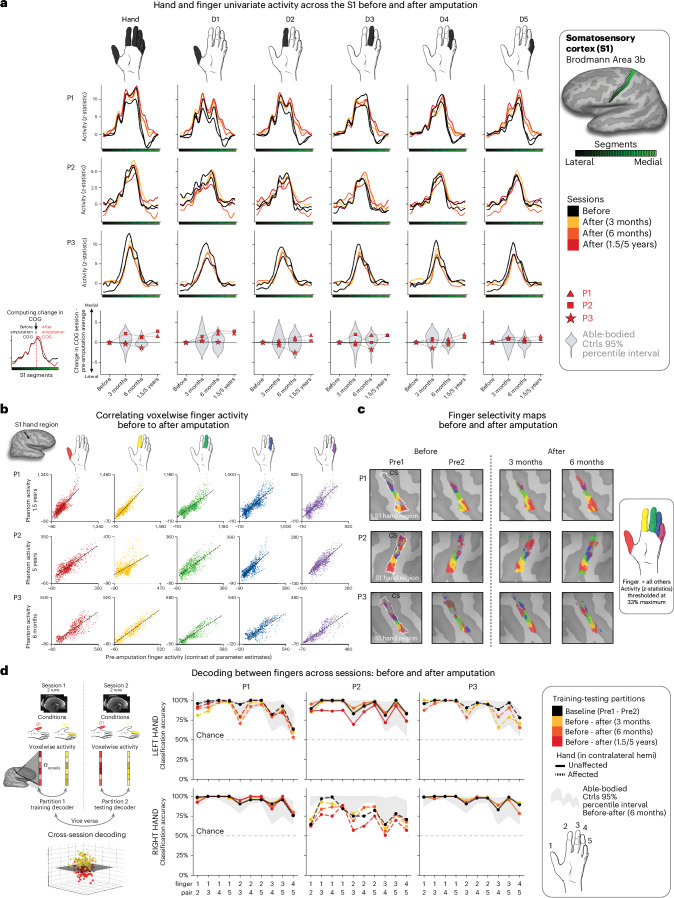
Stable hand representation in the affected hemisphere despite amputation. **a**, Longitudinal hand and individual finger activity (versus rest) projected across the S1 (BA3b) region of interest (ROI) segmented into 49 segments of similar height. The affected hand’s activity over five sessions (indicated in the legend) for each of the case study participants who underwent an amputation is shown; the bottom row shows the finger COG shifts before and after amputation. The black lines reflect the activity before amputation, the yellow, orange and red lines after amputation. The COG shifts of the case study participants (red) for the hand and individual fingers fell within the distribution of Ctrls (gray; six comparisons per participant; two-tailed Crawford *t*-test: P1 (6 months): 0.14 ≤ *P*_uncorr_ ≤ 0.58; P2 (6 months): 0.06 ≤ *P*_uncorr_ ≤ 0.81; P3 (6 months): 0.10 ≤ *P*_uncorr_ ≤ 0.91). Positive values indicate medial shifts (toward the feet); negative values indicate lateral shifts (toward the lips) in S1. Ctrl 95% percentile interval data are shown as gray violin plots. P1 data are shown as a red triangle. P2 data are shown as a red square. P3 data are shown as a red star. For simplicity, the Ctrl values are all for the left (nondominant) hand. **b**, Before and after amputation single-finger multivoxel correlations: for each finger of the case study participants, voxelwise activity correlations before and at the final scan after amputation are shown. All other correlations are comprehensively reported in Extended Data Fig. 5. The before to after amputation correlations for all participants were statistically significant (five two-tailed Pearson correlations per participant; P1 (6 months): 0.68 ≤ *r* ≤ 0.90, *P*_uncorr_ < 0.001; P2 (6 months): 0.80 ≤ *r* ≤ 0.85, *P*_uncorr_ < 0.001; P3 (6 months): 0.88 ≤ *r* ≤ 0.91, *P*_uncorr_ < 0.001). **c**, Finger selectivity maps before and after amputation. Each contrast map reflects the selective activity for each finger (versus all others), masked to the hand ROI. Each mask was minimally thresholded at 33% the maximum *z*-statistic and binarized. Color codes are indicated on the right. To visualize the multi-finger activity at a single voxel, a 70% opacity filter was applied to all finger maps. **d**, Left, Graphic illustration of multivoxel analyses using a linear SVM decoder. Right, Longitudinal classifier performance. The line colors denote training-testing cross-validation session pairs, respectively, as indicated in the legend. The gray-shaded area reflects the data of able-bodied Ctrls before and after (6 months) (95% percentile interval). Training the classifier on the pre-amputation data and testing it on the post-amputation data (and vice versa) revealed significantly above chance classification accuracies for all case study participants at all post-amputation sessions (two-tailed, one-sample *t*-test: P1: before 1.5 years: 89%; *P* < 0.001; P2: before 5 years: 67%; *P* < 0.001; P3: before 6 months: 88%; *P* < 0.001). All other annotations are depicted in Fig. 1.

**Extended Data Fig. 2:**
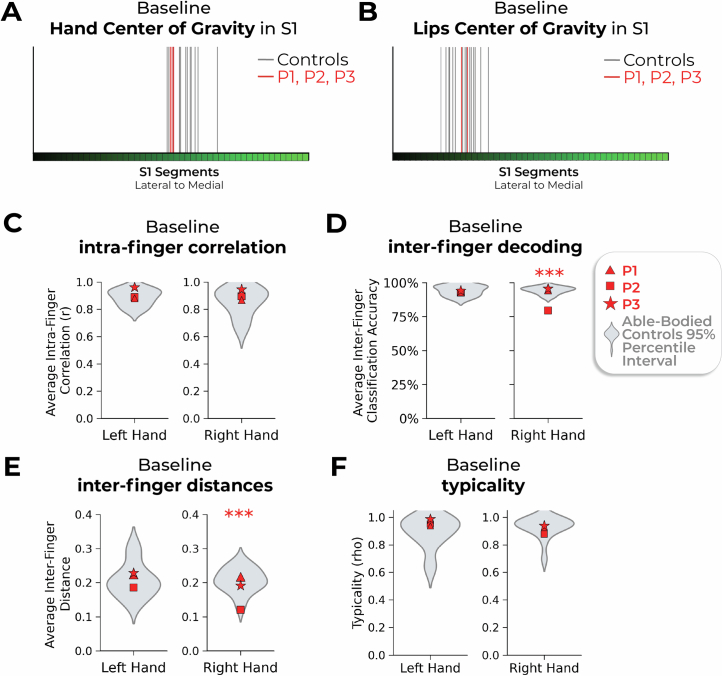
Baseline measures for the case-study participants that underwent an amputation versus able-bodied controls. Across all panels, we only report statistics when significant. Case-study participants showed similar responses to able-bodied controls in the baseline (pre-amputation) S1 center of gravity for the (**a**) hand and (**b**) lips. (**c**) All case-study participants had similar average intra-finger correlations between the two pre-sessions as controls. For baseline average inter-finger (**d**) classification accuracy and (**e**) distances. One case-study participant exhibited lower values for their affected hand only, relative to controls [Crawford t-test: decoding and distances: P2: p < 0.001] (**f**) All case-study participants had similar hand typicality between the two pre-sessions as controls. All other annotations the same as described in Figs. 2 and 3.

**Extended Data Fig. 3:**
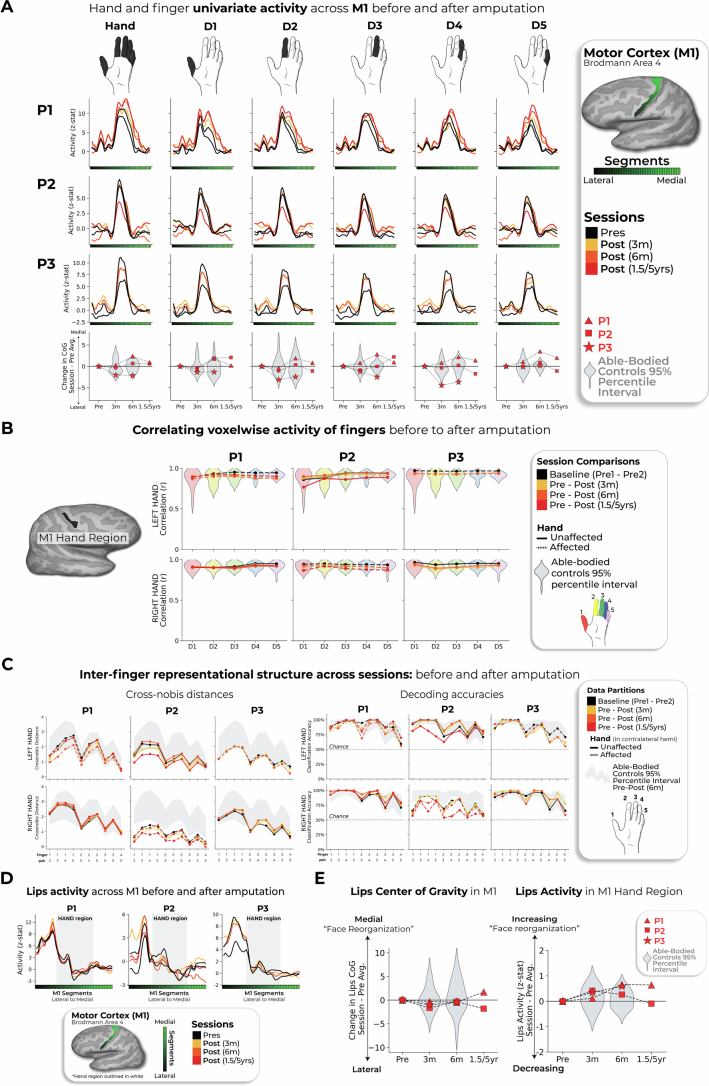
Replication of all primary results within motor cortex. (**a**) Hand and finger univariate activity across M1 before and after amputation. When testing the stability of the whole hand condition across sessions, all case-studies fell within the distribution of controls at all timepoints. (**b**) When correlating voxel wise finger activity across sessions, all case-studies exhibiting similar correlation coefficients as controls, for all fingers. Please refer to the Extended Data Fig. 5 caption for a more detailed understanding of the correlation analysis. (**c**) Inter-finger representational structure across sessions, measured using cross-nobis distances (left) and decoding accuracies (right). First, when assessing for atypicality in our case-studies pre-amputation compared to controls, only case-study P2 exhibited reduced average finger selectivity pre-amputation based on the RSA (Crawford t-test: t(15) = -3.15, p = 0.007) and decoding (t(15) = −3.9, p = 0.001; similar to what was observed in S1). Next, when testing for reductions in average finger selectivity at the 6-month timepoint, relative to baseline, only case-study P1 exhibited a significant reduction compared to controls [cross-nobis distances: 3 comparisons; t(15) = 2.33; puncorr=0.02); decoding: 3 comparisons; t(15) = 2.32; puncorr=0.03]. However, it returned to the typical range when later assessed at the 1.5 year timepoint (for both measures). We also noted that case-study P3 showed a significant reduction at the 6-month timepoint, relative to controls, in the decoding (3 comparisons; t(15) = 2.18, puncorr=0.046), but not the cross-nobis. (**d**) Lips univariate activity plotted across M1 before and after amputation. (**e**) All case studies showed typical session to session variability as controls in (left side) the lips center of gravity across M1 and (right side) lips activity in the M1 hand region. All annotations are the same as described in the captions of the Figs. 2–3 and Extended Data Fig. 5. Across all panels, we only report statistics when significant.

**Extended Data Fig. 4:**
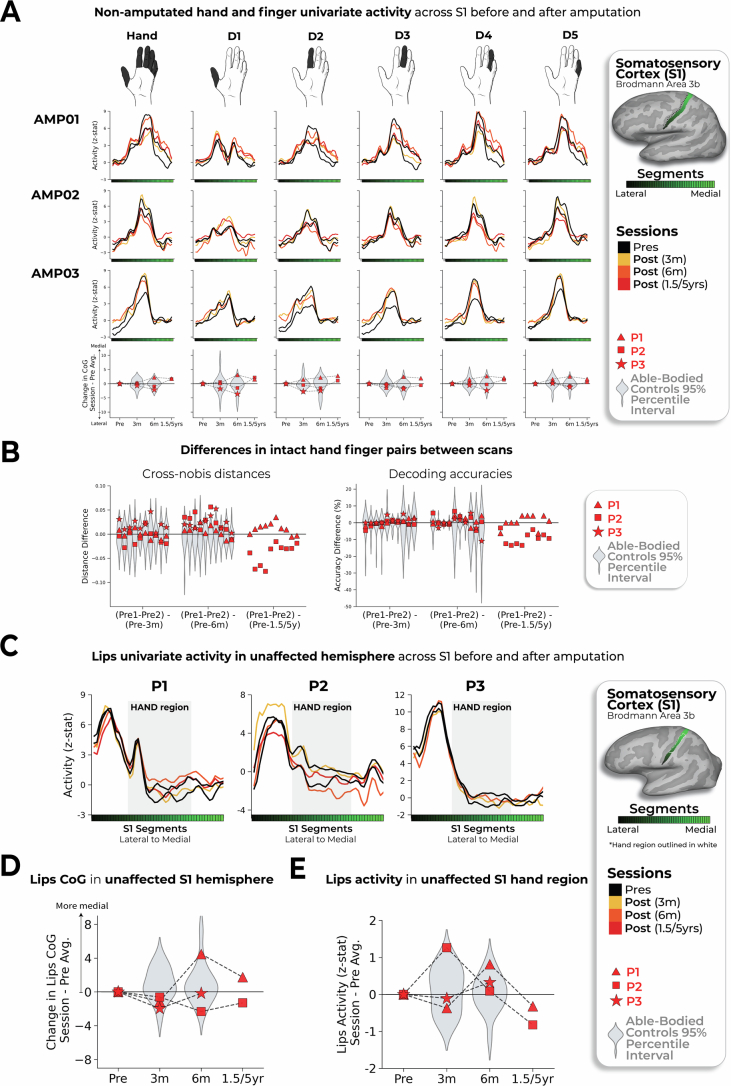
Stability of the intact (non-amputated) hand and lip topography in the non-affected hemisphere across amputation. (**a**) Intact hand and finger univariate activity across S1 before and after amputation. When testing the stability of the whole hand condition across sessions, all case-studies fell within the distribution of controls at all timepoints. (**b**) Unaffected (intact) hand between-session differences in inter-finger values. Difference values are depicted for the (left) cross-validated distances and (right) decoding accuracies. Classification/distance differences before and after amputation are visualized for each finger pair [Pre1-Pre2] minus [Pre Avg. – Post1 (3 m)] minus, [Pre1-Pre2] minus [Pre Avg. – Post2 (6 m)] and [Pre1-Pre2] minus [Pre Avg. – Post3 (1.55/y)]. Each violin plot reflects an individual finger pair (same order of finger-pairs as detailed in Fig. 2d). For consistency, the control values are all for the left-hand. When computing the session-to-session differences relative to controls, all case-study participants showed typical session-to-session variability in finger selectivity at the 6-month timepoint, relative to controls. (**c**) Longitudinal lips univariate in the unaffected hemisphere (contralateral to intact hand) across S1 before and after amputation. (**d**) All case study participants showed typical changes in the lips center of gravity (CoG) in the unaffected S1 hemisphere across scans, relative to controls. (**e**) When testing for changes in lip activity (in the unaffected hand region), one case-study, P1, exhibited a significant atypical increase in lip activity relative to controls at the 6-month timepoint (Crawford t-test: t(15) = 2.75, puncorr=0.01). However, the activity returned into the distribution of controls when tested at the 1.5 year timepoint (t(15) = 0, puncorr=0.99). All other annotations are the same as described in Figs. 2 and 3. We only report statistics when significant.

During scanning, participants performed visually cued movements involving tapping individual fingers, pursing lips and flexing toes. Case study participants demonstrated strikingly consistent hand and lip cortical maps before and after amputation (Fig. 1d). Projecting hand and individual finger activity profiles across S1 revealed stable activity before and after amputation, with phantom activity resembling the amplitude and spatial activity spread before amputation (Fig. 2a). A center of gravity (COG) analysis of these profiles revealed spatially consistent hand and individual finger activity in our case studies, with similar pre- and post-amputation session differences over 6 months as Ctrls (six Crawford *t*-tests per participant; P1: 0.14 ≤ *P*_uncorr_ ≤ 0.58; P2: 0.06 ≤ *P*_uncorr_ ≤ 0.81; P3: 0.10 ≤ *P*_uncorr_ ≤ 0.91). Notably, this stability could not be attributed to a pre-existing baseline difference as hand activity before amputation was normal relative to Ctrls (Extended Data Fig. 2a). Similar pre- and post-amputation stability was observed in the motor cortex (M1) (Extended Data Fig. 3a) and for the intact (unaffected) hand (Extended Data Fig. 4a).

**Extended Data Fig. 5:**
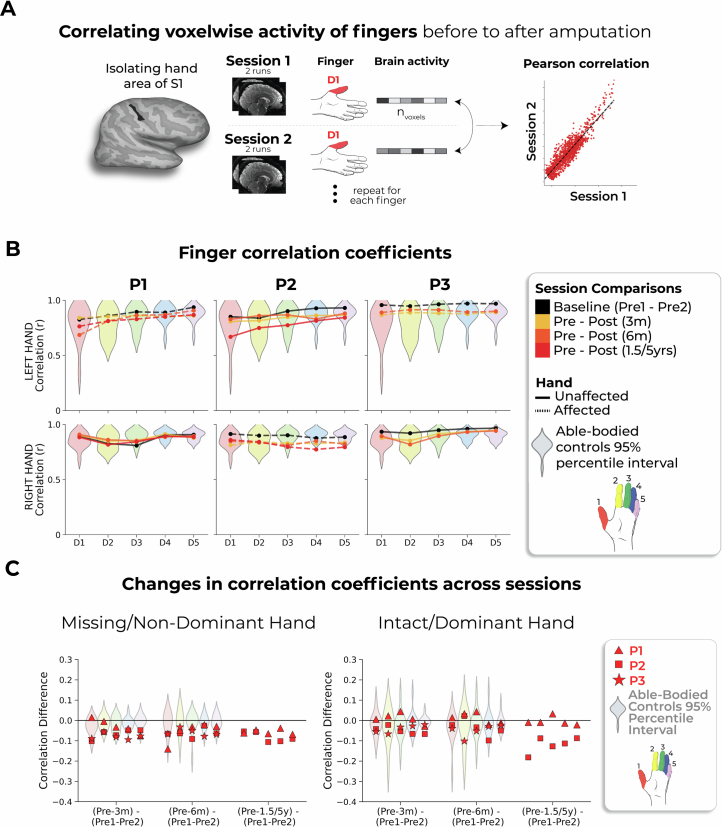
Correlating pre- to post-amputation multivoxel finger activity patterns. (**a**) Visualization depicting the inter-session Pearson correlations of individual fingers within the BA3b hand region. (**b**) Inter-session correlations for the left (top row) and right hands (bottom) in the contralateral hand ROI. Line colors indicate session pairings (indicated in the legend). For case-study participants, dashed line denotes the affected hand; solid line unaffected hand. Violin plots reflect able-bodied control’s Pre – Post (6 m) values. (**c**) Between-session differences in finger correlation coefficients. Difference values are depicted for the (left) missing or non-dominant hand of controls and (right) intact or dominant hand of controls. The difference values are ordered to reflect the increasing gap between sessions: [Pre1-Pre2] minus [Pre Avg. – Post1 (3 m)] minus, [Pre1-Pre2] minus [Pre Avg. – Post2 (6 m)] and [Pre1-Pre2] minus [Pre Avg. – Post3 (1.55/y)]. Each violin plot reflects an individual finger. When testing whether the case-study participants showed a unique reduction in the average correlation, across fingers, relative to controls, for the missing hand, only P3, at the 3-month timepoint, for the missing hand (not intact), showed a significant pre-post reduction in the average correlation coefficient, relative to controls (t(15) = −2.59, puncorr=0.02). However, this difference returned to the typical range of controls when later tested at the 6-month timepoint (t(15) = −1.23, puncorr=0.23). All other annotations are as in Fig. 2. We only report statistics when significant.

Next, we investigated the stability of S1 finger representation in greater detail using a multivoxel pattern analysis (Fig. 2b and Methods). Multivoxel activity patterns for the pre-amputated versus phantom fingers were significantly correlated at 6 months (five Pearson correlations per participant; P1: 0.68 ≤ *r* ≤ 0.90, *P*_uncorr_ < 0.001; P2: 0.80 ≤ *r* ≤ 0.85, *P*_uncorr_ < 0.001; P3: 0.88 ≤ *r* ≤ 0.91, *P*_uncorr_ < 0.001). Correlation coefficients at 6 months fell within the typical distribution seen in Ctrls (see Extended Data Fig. 5 and Supplementary Table 1 for the Ctrl values). Similar stability was observed in M1 (Extended Data Fig. 3b) and for the intact hand (Extended Data Fig. 4c). Combined, this confirmed that activity was largely stable before and after amputation at the single-voxel level.

We next considered finger selectivity, that is, the activity profiles for each finger versus the other fingers. Qualitative finger-mapping revealed preserved somatotopy before and after amputation (Fig. 2c). We applied a multivoxel pattern analysis using a linear support vector machine (SVM) classifier (Fig. 2d) to explore whether a pre-amputation-trained classifier could decode phantom finger movements (and vice versa). This analysis revealed significantly above chance classification for all case study participants across all post-amputation sessions (Fig. 2d; 2–3 one-sample *t*-tests per participant: P1 (before/1.5 years): 90%; *t*_(9)_ = 10.5, *P*_uncorr_ < 0.001; P2 (before/5 years): 67%; *t*_(9)_ = 4.85, *P*_uncorr_ < 0.001; P3 (before/6 months): 89%; *t*_(9)_ = 11.0, *P*_uncorr_ < 0.001), with similar evidence in M1 (Extended Data Fig. 3c).

**Extended Data Fig. 6:**
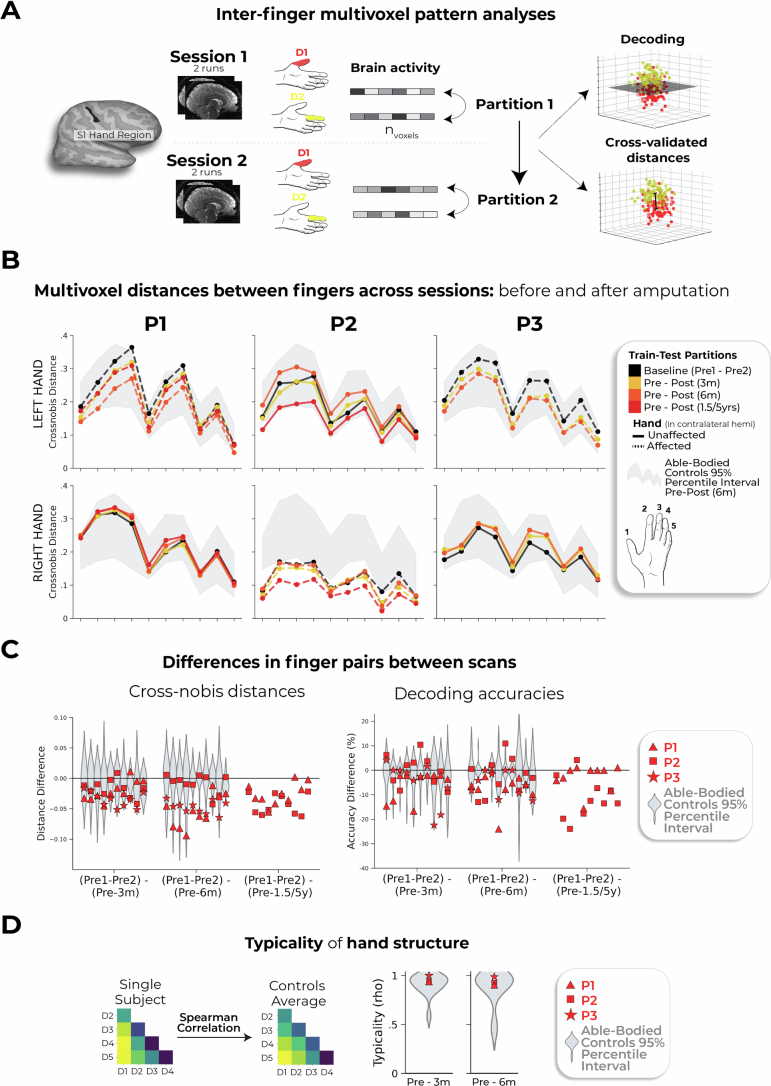
Representational similarity analysis of inter-finger representational structure. (**a**) Graphic illustration of multivoxel pattern analyses. (**b**) Inter-finger multivariate analysis using cross-validated Mahalanobis (cross-nobis) distances. Line colors denote train-test/cross validation session pairs, respectively as indicated in the legend. The gray shaded area reflects able-bodied control’s Pre – Post (6 m) data (95% percentile interval). (**c**) Classification/distance differences before and after amputation are visualized for each finger pair [Pre1-Pre2] minus [Pre Avg. – Post1 (3 m)] minus, [Pre1-Pre2] minus [Pre Avg. – Post2 (6 m)] and [Pre1-Pre2] minus [Pre Avg. – Post3 (1.55/y)]. Each violin plot reflects an individual finger pair (same order of finger-pairs as detailed in **b**). When comparing differences relative to controls, we observed some temporary, idiosyncratic reductions in average finger selectivity, relative to controls. First for the cross-nobis results, P1 showed a temporary reduction in average finger selectivity at 6 months (3 comparisons; t(15) = −2.79, puncorr=0.01), though later offset to the typical range at their follow-up 1.5-year scan. P2 only exhibited reduced selectivity only at the 5-year timepoint, though reduction seen in the intact hand as well (Extended Data Fig. 4). Finally, P3 exhibited reduced selectivity at 6 months relative to controls (2 comparisons; t(15) = −2.36, puncorr=0.03). For the decoding results, P2 seemed to show significantly reduced selectivity at the 5-year timepoint, though also reduced for the intact hand (Extended Data Fig. 4). (**d**) The representational typicality of the hand structure was estimated by correlating each session’s cross-validated Mahalanobis distances for each participant to a canonical inter-finger structure (controls average). All case-study participant’s typicality values fell within the distribution of controls. All other annotations are as in Fig. 2. We only report statistics when significant.

We next investigated whether amputation reduces finger-selective information, as suggested by previous cross-sectional studies^22^. Assessing for abnormalities in the pre-amputation data, we noted that one of the case study participants, P2, exhibited lower classification for the pre-amputation hand relative to Ctrls (Extended Data Fig. 1), probably because of P2’s impaired motor control before amputation (Supplementary Video 2). Our key question remains whether this information degrades further after amputation. When comparing selectivity differences over 6 months relative to Ctrls, none of the case study participants showed significant reductions in average finger selectivity (Crawford *t*-test: P1: *t*_(15)_ = −0.34, *P* = 0.73; P2: *t*_(15)_ = −0.24, *P* = 0.80; P3: *t*_(15)_ = −1.0, *P* = 0.33; Extended Data Fig. 6c). While finger selectivity was reduced at P2’s and P3’s final scan relative to their baseline (Fig. 2d; three Wilcoxon rank-sum tests per participant: P1 (1.5 years): *W* = 3.0, *P*_uncorr_ = 0.11; P2 (5 years): *W* = 2.0, *P*_uncorr_ = 0.005; P3 (6 months): *W* = 1.0, *P*_uncorr_ = 0.01), these reductions could be attributed to the much greater longitudinal variability between training and testing classifier samples^23^. To further explore this, we directly compared the finger selectivity of the affected hand versus the unaffected hand. For two of three of our participants, at the 6-month time point, we observed decreased finger-selective information in the affected hand relative to the unaffected hand, compared with Ctrls (dominant hand versus non-dominant hand; two Crawford *t*-tests per participant; before 6 months: P1: *P*_uncorr_ = 0.03; P2: *P*_uncorr_ = 0.03; P3: *P*_uncorr_ = 0.10; Supplementary Fig. 1). Collectively across analyses, the decoding results suggested slight (uncorrected) reductions in finger selectivity or increased finger selectivity for the intact hand.

We also performed a complementary representational similarity analysis (RSA) using Mahalanobis distances (a continuous measure of finger selectivity), cross-validated across sessions. Like the decoding, RSA confirmed that finger-selective information was significantly consistent across amputation for all case study participants at all post-amputation time points (2–3 one-sample *t*-tests per participant: *P*_uncorr_ < 0.0001; Extended Data Fig. 6a,b), with similar evidence in M1 (Supplementary Fig. 3c). We noted a few temporary, idiosyncratic (uncorrected) instances of reduced finger selectivity relative to Ctrls (Extended Data Fig. 6c). Using the RSA distances, we also tested the typicality of the inter-finger representational structure, an additional feature of hand representation. Correlating each participant’s inter-finger pattern to a canonical pattern revealed no deterioration in typicality scores 6 months after amputation compared to Ctrls, with P3 even showing higher typicality than the Ctrl group (Crawford *t*-test: P1: *t*_(15)_ = −0.9, *P* = 0.38; P2: *t*_(15)_ = −0.9, *P* = 0.38; P3: *t*_(15)_ = −3.5, *P* = 0.003; Extended Data Fig. 6d). Therefore, despite idiosyncratic reductions in finger selectivity, the representational structure was preserved after amputation.

**Fig. 3:**
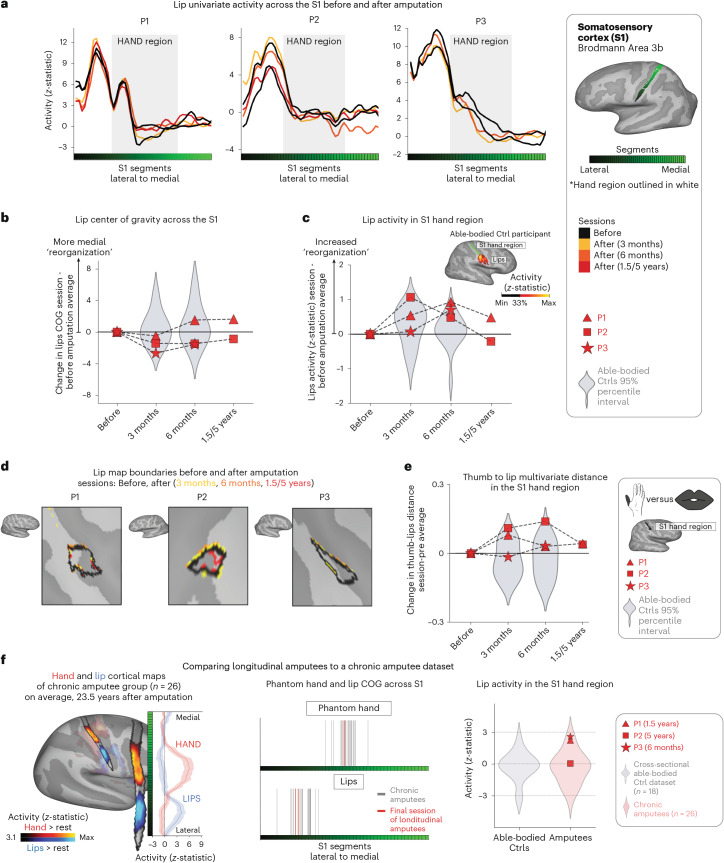
No evidence for lip reorganization after amputation. **a**, The lip activity (versus rest) of each case study participant for their sessions projected across the S1 ROI. The black lines reflect pre-amputation activity, with the yellow (3 months), orange (6 months) and red (1.5/5 years) lines reflecting activity after amputation. The gray region depicts the approximated coverage of the hand portion in the S1. **b**, All case study participants showed typical longitudinal variability at their 6-month scan, relative to Ctrls, for the lip COG. Positive values reflect medial shifts (toward the hand). **c**, All case study participants showed typical lip activity in the S1 hand region at the final scan. The right corner depicts representative Ctrl participant activity for the lips (versus the feet) minimally thresholded at 33% the maximum *z*-statistic. **d**, All case study participants exhibited no expansion of the lip map boundaries toward the hand region. Maps were masked to the S1 ROI and were minimally thresholded (*z* > 4.5). **e**, All case study participants showed stable thumb-to-lip multivariate Mahalanobis distances cross-validated at their final scan, relative to Ctrls. **f**, Comparing the case study participants to a chronic amputee dataset (*n* = 26). Left, Chronic amputee’s group-level cortical activation maps of the phantom hand and lips (versus rest) projected onto a single hemisphere (minimally thresholded at *z* > 3.1). Opacity was applied to activity outside the S1 ROI. Group univariate activity was plotted as a line (group mean ± s.e.) for the phantom hand (red) and lips (blue) across the S1 ROI. Middle, All case study participants, relative to chronic amputees, showed a typical COG for both the phantom hand (top) and lips (bottom). Right, All case study participants exhibited typical lip activity in the S1 hand region during their final session, which is consistent with chronic amputees. The magnitude of lip activity (95% percentile interval) in the S1 hand region for a secondary able-bodied Ctrl group (*n* = 18) is shown in gray. Chronic amputees are shown in light red and the last session data for the case study participants are shown in dark red. All other annotations are the same as described in Fig. 2.

**Extended Data Fig. 7:**
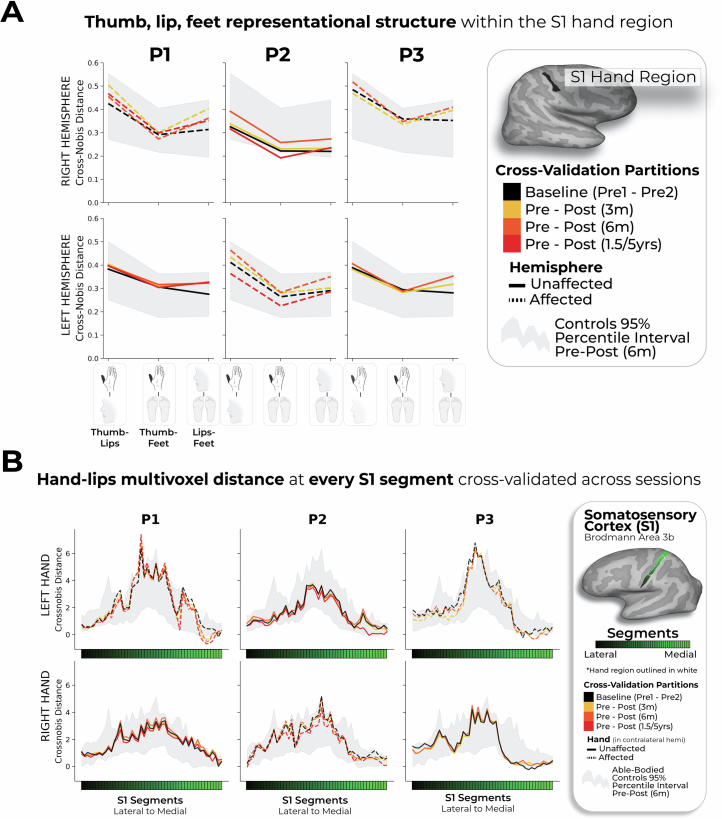
Thumb, lip and feet distances within the S1 hand region. (**a**) Multivariate distances between the thumb, lip and feet cross-validated across sessions depicted for the right (top row) and left hemisphere (bottom) of the case-study participants that underwent an amputation and controls, contralateral to the thumb side being moved. Distances appear in the following order: (1) thumb-lips, (2) thumb-feet, (3) lips-feet. Line colors indicate session pairings (indicated in the legend). For case-study participants, dashed line denotes the affected hemisphere; solid line unaffected hemisphere. Grey shaded area reflect able-bodied control’s Pre – Post (6 m) values. For the affected hemisphere of the case-study participants, all distances fell within the typical range of the able-bodied controls. (**b**) We also tested whether changes occurred in the multivariate hand-lip distance when performed within each of the 49 S1 segments/ All case-study participants showed similar distances across sessions, before and after amputation. All other annotations are the same as described in Fig. 2.

Finally, we examined changes in lip representation, previously implicated with reorganization after arm amputation^4,7^. Projecting hand and lip univariate activity onto the S1 segments revealed no evidence of lip activity shifting into the hand region after amputation (Fig. 3a). All case study participants showed typical longitudinal variability at their 6-month scan, relative to Ctrls, for lip COG (Fig. 3b; Crawford *t*-test: P1: *t*_(15)_ = 0.25, *P* = 0.80; P2: *t*_(15)_ = −0.89, *P* = 0.38; P3: *t*_(15)_ = −0.9, *P* = 0.37). Furthermore, lip activity in the S1 hand region at the final scan was typical (Fig. 3c; P1 (1.5 years): *t*_(15)_ = 0.8, *P* = 0.20; P2 (5 years): *t*_(15)_ = −0.5, *P* = 0.71; P3 (6 months): *t*_(15)_ = 1.2, *P* = 0.10). Also, when visualizing the lip map boundaries within S1 for all sessions, using a common minimum threshold, there was no evidence for an extension of the lip map (Fig. 3d). Examining the multivariate lip representational content, P2 showed an increased lip-to-thumb multivariate distance at their 6-month scan, relative to Ctrls (Fig. 3e; Crawford *t*-test: P1: *t*_(15)_ = 0.69, *P* = 0.25; P2: *t*_(15)_ = 3.1, *P* = 0.003; P3: *t*_(15)_ = 0.74, *P* = 0.23; intact hand and feet data are included in Extended Data Fig. 7) However, it returned to the typical range of Ctrls when assessed at their 5-year time point. Similar stability was found in M1 (Extended Data Fig. 3) and the unaffected hemisphere (Extended Data Fig. 4). These results demonstrate that amputation does not affect lip topography or representational content in S1.

**Extended Data Fig. 8:**
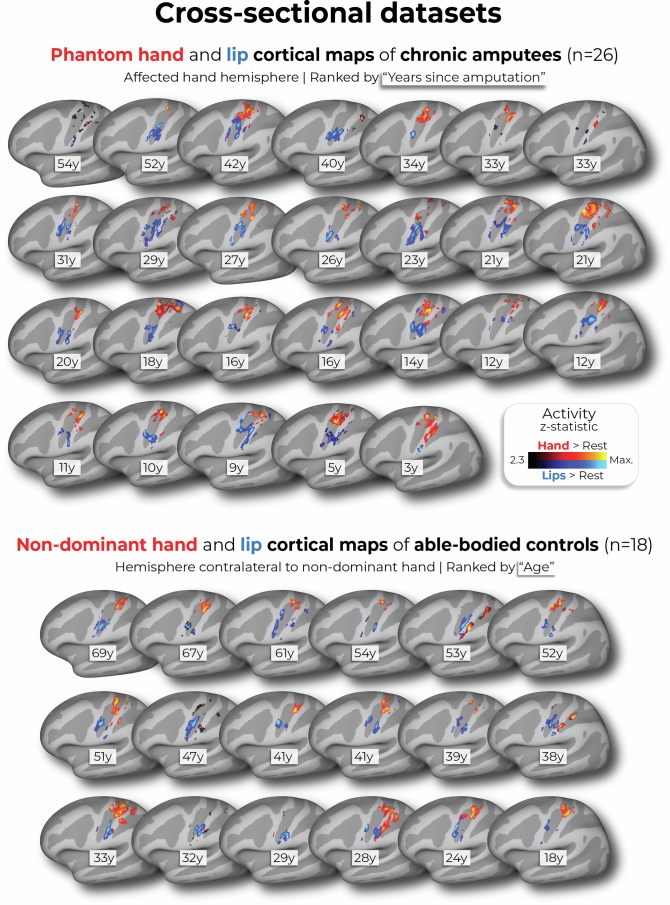
Hand and lip cortical maps of cross-sectional datasets. Participant hand and lip cortical maps – registered to a standard cortical surface – are visualized for the chronic amputee participants (top row; n = 26) and secondary able-bodied control participants who underwent the same procedures as the chronic amputees (n = 18; bottom row). Hand maps for the amputees reflect moving their phantom hand, while for controls reflect moving their non-dominant hand (in the contralateral hemisphere). All maps are contrasted against rest, minimally thresholded at 50% the maximum z-statistic and masked to Brodmann regions: 1, 2, 3a, 3b, and 4. Amputee maps are ranked by the numbers of years since amputation at the time of the scan and control maps are ranked by the participants age at the time of the scan.

To complement our longitudinal findings, we compared our case studies to a cohort of 26 chronic upper-limb amputee participants, on average 23.5 years after amputation (Fig. 3f; individual hand and lip cortical maps shown in Extended Data Fig. 8). The topographical features of our case studies were comparable to chronic amputees for both the phantom hand [Crawford *t*-test: P1 (1.5 years): *t*_(15)_ = 0.28, *P* = 0.77; P2 (5 years): *t*_(15)_ = 0.29, *P* = 0.77; *P* = 0.77; P3 (6 months): *t*_(15)_ = 0.28, *P* = 0.22; *P* = 0.82] and lips [P1 (1.5 years: *t*_(15)_ = 0.53, *P* = 0.59; P2 (5 years): *t*_(15)_ = 0.01, *P* = 0.98; P3 (6 months): *t*_(15)_ = 0.37, *P* = 0.71]. Average lip activity within the S1 hand region was slightly (although not significantly) higher for a few of our case studies relative to chronic amputees [Crawford *t*-test: P1 (1.5 years): *t*_(15)_ = 1.6, *P* = 0.10; P2 (5 years): *t*_(15)_ = 0.24, *P* = 0.81; P3 (6 months): *t*_(15)_ = 1.8, *P* = 0.065], reflecting that lip activity does not steadily increase in the years after amputation. Collectively, these results provide long-term evidence for the stability of hand and lip representations despite amputation.

Beyond the stability of lip representation across amputation, our findings reveal highly consistent hand activity despite amputation. This unchanged hand representation challenges the foundational assumption that S1 activity is primarily tied to its peripheral inputs, suggesting S1 is not a passive relay of its peripheral input, but an active supporter of a resilient ‘model’ of the body, even after amputation. Therefore, we conclude that, in the adult brain, S1 representation can be maintained by top-down (for example, efferent) inputs. This interpretation sheds new light on previous studies showing similar S1 topographical patterns activated by touch^24^, and executed^25^ and planned movement^26^.

**Extended Data Fig. 9:**
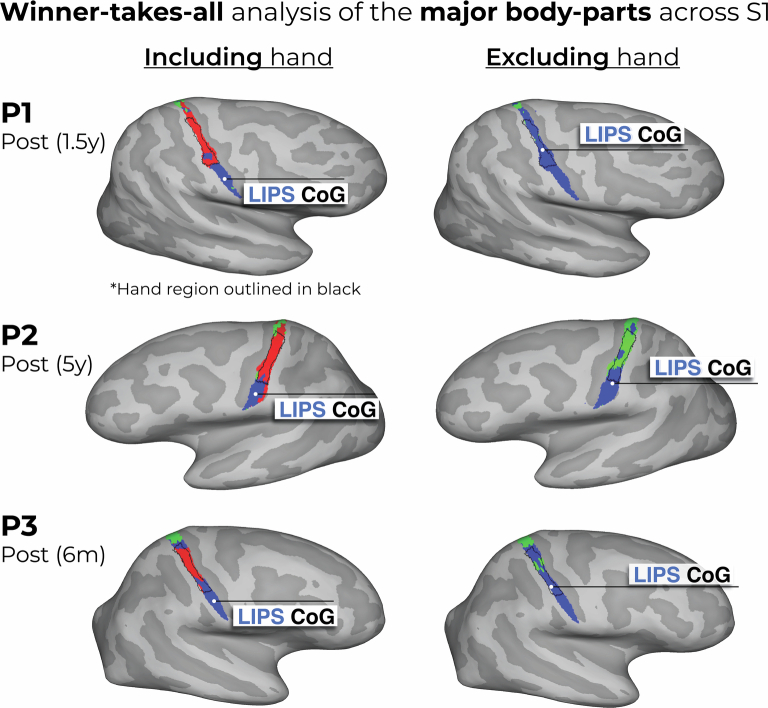
Winner-takes-all analysis of the major body parts (hand, lips and feet) across S1. Using the data from the last session of each participant, each voxel was awarded to the body-part with the highest response. Left column – we show the winner-takes-all analysis when performed on 3 body-parts: hand (red), lips (blue) and feet (green) versus (Right column) when excluding the physically absent hand. This comparison reveals supposed large-scale expansions of the lips or feet into the deprived hand region (black outline) post-amputation. We’ve also depicted the center of gravity (CoG) of the winner-takes-all lip cluster (white circles) to further demonstrate this. When excluding the hand activity, the CoG of the lips ‘shifts’ towards the hand area. Thus, ignoring the primary body part – depending on your analysis choices – can substantially bias the results^61,62^. Combined with the use of cross-sectional designs, this analysis approach has led to the impression of cortical remapping and even large-scale reorganization of the lip representation following amputation. Crucially, the newly assigned winner in the hand area [left panel] has rarely been directly compared against the persistent representation of the missing hand, and indeed, indicative evidence show that this recorded activity in the hand area is weak (we extensively discuss this in our recent review ref. ^17^).

Because of the limitations of nonhuman models that cannot communicate phantom sensations, it is not surprising that the persistent representation of a body part, despite amputation, has been neglected in previous studies. Without access to this subjective dimension, researchers may have missed the profound resilience of cortical representations. Instead, previous studies determined S1 topography by applying a ‘winner-takes-all’ strategy, probing responses to remaining (intact) body parts and noting the most responsive body parts in the input-deprived cortex. Ignoring phantom representations in these analyses leads to severe biases in the interpretation of the area’s inputs (as demonstrated in Extended Data Fig. 9). Combined with cross-sectional designs, this has incorrectly led to the impression of large-scale reorganization of the lip representation after amputation. Our longitudinal approach reveals no signs of topographic reorganization in S1, not even subtle upregulation from homeostasis, further reinforcing the notion that S1 is not governed by deprivation-driven plasticity.

For brain–computer interfaces, our findings demonstrate a highly detailed and stable representation of the amputated limb for long-term applications^27^. For phantom limb pain treatments, our study indicates that targeted muscle reinnervation and regenerative peripheral nerve interfaces do not ‘reverse’ reorganization or alter the cortical hand representation^22,28^. Finally, our findings affirm the unaltered nature of adult sensory body maps after amputation, suggesting that Hebbian and homeostatic deprivation-driven plasticity is even more marginal than considered by even the field’s strongest opponents of large-scale reorganization^17,29^.

## Methods

Our key methodology involved longitudinal comparisons across amputation. This approach was designed to overcome known limitations in cross-sectional designs, where inter-participant variability could spuriously influence group comparisons, particularly when considering small group sample sizes or small effects. An important additional consideration regarding reorganization research in amputees is the difficulty to interpret whether sensorimotor activity for the missing (phantom) hand reflects preserved representation (that is, whether it reflects the same representational attributes as the physical hand before amputation), or an altered hand representation, which exhibits canonical hand representation features, albeit distinct from the pre-amputation hand. The main limitation of longitudinal designs is the contribution of any time-related effects, for example, because of changes in magnetic resonance scanning hardware^30^ or participants’ experience (for example, familiarity with the study environment^31^), which are not directly related to the amputation. To account for nonrelated variables, we also scanned our case studies and Ctrl participants over a similar time frame. For two of our case studies, we had an opportunity to follow up on our procedures after an extended period (1.5/5 years after amputation). As this was not planned in the original design, we were unable to obtain related time points in our Ctrls. Therefore, all comparisons to the Ctrl cohort are focused on the 6-month post-amputation time point.

### Participants

#### Longitudinal case study participants who underwent an amputation

Over a 7-year period and across multiple NHS sites in the UK, we recruited 18 potential participants preparing to undergo hand amputations. Because of many factors (for example, MRI safety contraindications, no hand motor control, age outside the ethics range, high level of disability), we could only perform pre-amputation testing on six volunteers. Because of additional factors (complications during surgery, general health, retractions), we successfully completed our full testing procedure on three participants (for participant demographics, see Extended Data Table 1).

Pre-amputation scans for P1 and P2 were collected 24 h apart and within 2 weeks of their amputations. P3 had a 2.5-year gap between the pre-amputation scans due to coronavirus disease-related delays in testing and in scheduling uncertainty related to their amputation surgery. Their amputation surgery took place 3 months after their second pre-amputation scan.

#### Case study participant amputation surgeries

There are noteworthy differences in the amputation surgeries of the three case study participants. P1 underwent an amputation to combat a rapidly developing arteriovenous malformation in the upper arm. Before amputation, they had a relatively high level of motor control in the pre-amputation hand. Additionally, P1’s amputation included more advanced surgical techniques, involving a combination of targeted muscle reinnervation^32^ and regenerative peripheral nerve interfaces^33^. In these approaches, rather than simply cutting the residual nerve, the remaining nerves were sutured to a new muscle (targeted muscle reinnervation) or implanted with a nerve graft (regenerative peripheral nerve interface) (in P1’s case, the technique varied depending on the muscle; Supplementary Fig. 2). P2 underwent a traditional amputation procedure to remove a sarcoma tumor that had been slowly progressing since 1995. Multiple surgeries of the arm, before the amputation, left them with restricted motor control of the fingers, although still able to move them (Supplementary Video 2). Similarly, P3 was diagnosed with Severell–Martorell syndrome, which had led to their left arm having multiple chronic bone fractures. They underwent a traditional amputation procedure, where the major nerves were left to naturally retract. It is important to note that the diversity of conditions, procedures and postoperative states across our case studies strengthen the universality of our results, which were consistent across case studies.

#### Longitudinal able-bodied Ctrl group

In addition to the case study participants who underwent an amputation, we tested a Ctrl group that included 16 older able-bodied participants (nine females; mean age ± s.d. = 53.1 ± 6.37; all right-handed). The Ctrl group also completed four functional MRI (fMRI) sessions at the same timescale as the participants who underwent an amputation and were age-matched to P2 and P3. Four additional Ctrls were recruited for this group; however, we did not complete their testing because of dropout and incidental findings captured during the MRI sessions.

Ethical approval for all longitudinal study participants was granted by the NHS National Research Ethics Committee (no. 18/LO/0474) and in accordance with the Declaration of Helsinki (v.2013). Written informed consent was obtained from all participants before the study for their participation, and for data storage and dissemination.

#### Cross-sectional datasets

From three previous studies (one unpublished study and refs. ^14,34^), we pooled two cross-sectional fMRI datasets: (1) a group of chronic amputees (*n* = 26) and (2) a secondary group of able-bodied Ctrls (*n* = 18). The chronic amputee group included 26 upper-limb amputee participants (four females; mean age ± s.d. = 51.1 ± 10.6; 13 missing the left upper-limb; level of amputation: 17 transradial, eight transhumeral and one at the wrist; mean years since amputation ± s.d. = 23.5 ± 13.5). The secondary able-bodied Ctrl group included 18 able-bodied participants (seven females; mean age ± s.d. = 43.1 ± 14.62; 11 right-handed). For more information on these datasets, see the Supplementary Methods (https://osf.io/s9hc2/).

#### Longitudinal younger adult able-bodied Ctrl dataset

P1 is younger than the longitudinal Ctrl group. As such, we reanalyzed a previously collected dataset including 22 able-bodied Ctrls of a similar age to P1 (mean ± s.d. = 23.2 ± 3.8); each were scanned twice, 1 week apart on the same fMRI task and scanner^35^.

### Questionnaires

Because of a restricted time window for performing the tests before amputation, and the participants’ high level of physical discomfort and emotional distress, we were highly limited in the number of assessments we could perform. As such, we primarily focused on the functional neuroimaging tasks. However, in addition, we collected data on multiple questionnaires and had participants perform a functional ecological task.

#### Kinesthetic vividness

Kinesthetic vividness was quantified for each finger before and after the amputation (When moving this finger, how vivid does the movement feel? Please rate between 0 (I feel no finger movement) to 100 (I feel the finger movement as vividly as I can feel my other hand finger moving)).

#### Finger motor control

Perceived finger movement difficulty was quantified for each finger before and after amputation (When moving this finger, how difficult is it to perform the movement? Please rate between 100 (I found it as easy as moving the homologous finger in the unimpaired hand) to 0 (the most difficult thing imaginable)).

#### Pain ratings

Before and after amputation, case study participants were asked to rate the frequency of their pre-amputation limb pain or post-amputation phantom limb pain, respectively, as experienced in the last year, as well as the intensity of the worst pain experienced during the last week (or in a typical week involving pain; Extended Data Table 1). Chronic pain was calculated by dividing the worst pain intensity (scale 0–100: ranging from no pain to worst pain imaginable) by pain frequency (1, all the time; 2, daily; 3, weekly; 4, several times per month; and 5, once or less per month). This approach reflects the chronic aspect of pain because it combines both frequency and intensity^36,37^. A similar measure was obtained for painless phantom sensation vividness and stump pain. Participants also filled out the painDETECT questionnaire^38^. Additionally, before and after amputation, participants reported intensity values for different words describing different aspects of pain, quantified using an adapted version of the McGill Pain Questionnaire^39^. For each word, participants were asked to describe the intensity between 0 (nonexisting) to 100 (excruciating pain) as it related to each word. We used a larger response scale than standard to allow participants to articulate even small differences in their pain experience (Extended Data Fig. 1).

#### Functional index

Before and after amputation, case study participants were asked to rate their difficulty at performing a variety of functional activities because of their upper-limb problem, quantified using the Upper Extremity Functional Index^40^.

### Ecological task

To characterize habitual compensatory behavior, participants completed a task involving wrapping a present (based on ref. ^41^). Task performance was video-recorded but is not reported in this paper.

### Finger movement task

To qualitatively capture how participants moved when cued to perform individual finger movements, at each session, we asked participants to perform a finger movement task where we cued them to move a single finger. Case study participants were cued to perform unilateral movements of the phantom fingers and intact fingers, and then mirrored the movements of the intact and phantom fingers simultaneously. Task performance was video-recorded and is shown in Supplementary Video 2.

### Intact finger kinematic task

To test whether the intact fingers were being moved simultaneously during phantom finger movements, we invited two of the three case study participants back for a separate session to assess the kinematics of the intact fingers. The task setup and data are shown in Supplementary Fig. 4.

### Scanning procedures

Each MRI session for the longitudinal cohort consisted of a structural scan, four fMRI finger-mapping scans and two body localizer scans, which we report in this article. The additional cross-sectional datasets are detailed in the Supplementary Methods.

### fMRI task design

#### Finger-mapping scans

The fMRI design was the same as a previous study from our laboratory^35^, although specific adaptations were made to account for the phantom experience of the case study participants who underwent an amputation (described below). Considering that S1 topography is similarly activated by both passive touch and active movement^24^, participants were instructed to perform visually cued movements of individual fingers, bilateral toe curling, lips pursing or resting (13 conditions in total). This was performed using PsychoPy (v.2021.1.1). The different movement conditions and rest (fixation) cue were presented in 9-s blocks, each repeated four times in each scan. Additionally, each task started with 7 s of rest (fixation) and ended with 9 s of rest.

To simulate a phantom-like tactile experience for the participants before amputation, the affected hand was physically slightly elevated during scanning such that affected finger-tapping-like movements were performed in the air. Alternatively, for the unaffected hand (before and after amputation), the individual finger movements were performed as button presses on an MRI-compatible button box (four buttons per box) secured on the participant’s thigh. The movement of the thumb was performed by tapping it against the wall of the button box. For the Ctrl participants, half of the participants had the right hand elevated, performing the finger movements in the air, and the other half had the left hand elevated.

Instructions were delivered via a visual display projected into the scanner bore. Ten vertical bars, representing the fingers, flashed individually in green at a frequency of 1 Hz, instructing movements of a specific finger at that rate. Foot and lip movements were cued by flashing the words ‘Feet’ or ‘Lips’ at the same rate. Each condition was repeated four times in each run in a semi-counterbalanced order. Participants performed four scan runs of this task. One Ctrl participant was only able to complete three runs of the task for one of the sessions.

#### Imagery control scans

In each of the two body localizer scans, participants were visually cued to move each hand, imagine moving the affected (case study participants) or nondominant hand (Ctrls), in addition to actual lip, toe (on the affected side only) and arm (on the affected side only) movements. The different movement conditions and a rest (fixation) cue were presented in 10-s blocks and repeated four times in each scan.

### MRI data acquisition

MRI images were obtained using a 3T Prisma scanner (Siemens) with a 32-channel head coil. Anatomical data were acquired using a T1-weighted magnetization prepared rapid acquisition gradient echo sequence with the following parameters: repetition time (TR) = 2.53 s, echo time (TE) = 3.34 ms, field of view (FOV) = 256 mm, flip angle = 7 degrees and voxel size = 1-mm isotropic resolution. Functional data based on the blood-oxygenation-level-dependent signal were acquired using a multiband gradient echo-planar T2*-weighted pulse sequence^42^ with the following parameters: TR = 1.5 s, TE = 35 ms, flip angle = 70 degrees, multiband acceleration factor = 4, FOV = 212 mm, matrix size of 106 × 106 and voxel size = 2-mm isotropic resolution. Seventy-two slices, with a slice thickness of 2 mm and no slice gap, were oriented parallel to the anterior commissure–posterior commissure, covering the whole cortex, with partial coverage of the cerebellum. Each of the four functional runs comprising the main task consisted of 335 volumes (8 min 22 s). Additionally, there were 204 volumes for the two imagery control scans (5 min 10 s). For all functional scans, the first dummy volume of every run was saved and later used as a reference for coregistration.

### fMRI analysis

fMRI data processing was carried out using the FMRIB Expert Analysis Tool (FEAT v.6.0), part of FSL (the FMRIB Software Library, www.fmrib.ox.ac.uk/fsl), in combination with custom bash, Python (v.3) and MATLAB scripts (R2019b, v.9.7, MathWorks, including an RSA toolbox)^43,44^. Cortical surface reconstructions were produced using FreeSurfer v.7.1.1 (refs. ^45,46^) and the Connectome Workbench (https://humanconnectome.org/) software. Decoding analyses were carried out using scikit-learn v.1.2.2.

### fMRI preprocessing

The following prestatistical processing was applied: motion correction using MCFLIRT^47^, non-brain removal using BET^48^, spatial smoothing using a Gaussian kernel of full width at half maximum FWHM of 3 mm for the functional task data, grand-mean intensity normalization of the entire four-dimensional dataset by a single multiplicative factor and high-pass temporal filtering (Gaussian-weighted least-squares straight line fitting, with *σ* = 90 s). Time series statistical analysis was carried out using FILM with local autocorrelation correction^49^. The time series model included trial onsets convolved with a double gamma hemodynamic response function; six motion parameters were added as confound regressors. Indicator functions were added to model out single volumes identified to have excessive motion (>0.9 mm). A separate regressor was used for each high-motion volume (deviating more than 0.9 mm from the mean position). For the finger-mapping scans, the average number of outlier volumes for an individual scan, across all participants, was 1.5 volumes.

To ensure that all longitudinal sessions (Pre1, Pre2, 3 months, 6 months, 1.5/5 years) were well aligned for each participant, we calculated a structural mid-space between the structural images from each session, that is, the average space in which the images were minimally reorientated^50^. The functional data for each individual scan run in a session were then registered to this structural mid-space using FLIRT^47,51^.

### Low-level task-based analysis

We applied a general linear model (GLM) using FEAT to each functional run. For the primary task, the movement of each finger or body part (ten fingers, lips and feet, total of 12 conditions) was modeled against rest (fixation). To capture finger selectivity, the activity for each finger was also modeled as a contrast against the average activity of all other fingers of the same hand.

We performed the same GLM analysis on the six conditions of the imagery scans. To capture the selectivity of actual attempted phantom movements versus imagine phantom hand movements, the activity of the attempted hand movement was also modeled as a contrast against the imagined hand movement.

For each participant, parameter estimates of each of the different conditions (versus rest) and GLM residuals of all voxels were extracted from each run’s first-level analysis. All analyses were performed with the functional data aligned to the structural mid-space.

### ROIs

#### S1: Brodmann area 3b

We were specifically interested in testing changes in topography within (and around) Brodmann area 3b (BA3b). First, the structural mid-space T1 image were used to reconstruct the pial and white–gray matter surfaces using FreeSurfer’s recon-all. Surface coregistration across hemispheres and participants was conducted using spherical alignment. Participant surfaces were nonlinearly fitted to a template surface, first in terms of the sulcal depth map and then in terms of the local curvature, resulting in an overlap of the fundus of the central sulcus across participants^52^.

#### S1 (BA3b) hand ROI

The BA3b ROI was defined in the fsaverage template space using probabilistic cytotectonic maps^52^ by selecting all surface nodes with at least 25% probability of being part of the gray matter of BA3b^53^. Furthermore, for the multivoxel pattern analyses, we restricted the BA3b ROI to just the area roughly representing the hand. This was done by isolating all surface nodes 2.5 cm proximal or distal of the anatomical hand knob^54^. An important consideration is that this ROI may not precisely reflect BA3b for each participant and may contain relevant activity from neighboring S1 areas because of the nature of our data (3T fMRI, smoothing full width at half maximum 3 mm) and the probabilistic nature of the atlas. As such, we considered this as a definitive localizer of S1 and an indicative localizer of BA3b. Surface ROIs were then mapped to the participant’s volumetric high-resolution anatomy.

#### Forty-nine segments of the BA3b

To segment the BA3b into 49 segments, we loaded the fsaverage flattened cortical surface with the boundaries of the BA3b ROI, as defined by the Glasser atlas^55^. We rotated the map so that the central sulcus was perpendicular to the axis. We overlayed a box with 49 segments of equal height on this ROI. By masking the box to the ROI, we constructed the 49 segments of the BA3b ROI. Because this masking approach requires drawing boundary lines using the vertices on the cortical flat map, we could optimally only get 49 segments (maximum) without issues with the boundary drawing approach. These ROIs were then mapped onto the participant’s volumetric high-resolution anatomy and further to the participant’s cortical surfaces.

#### M1: Brodmann area 4

The approach for defining the motor cortex ROI was the same as described above, with the sole exception of selecting the Brodmann area 4 region.

### Projecting functional activity onto the cortical surface

Using the cortical surfaces generated using recon-all, fMRI maps were projected to the surface using the workbench command’s volume-to-surface mapping function, which included a ribbon-constrained mapping method. The cross-sectional datasets were the only exception, where we projected all maps onto a standard cortical surface (Supplementary Methods).

### Univariate activity

#### Contrast maps for moving versus imagine moving the phantom

To visualize the contrast maps for attempted versus imagine phantom hand movements, estimates from the two imagery control scan runs for the participant’s post-amputation (6-month) session were averaged in a voxelwise manner using a fixed-effects model with a cluster-forming *z*-threshold of 3.1 and family-wise error-corrected cluster significance threshold of *P* < 0.05. Maps were then projected onto each participant’s cortical surface. These contrast maps are visualized in Fig. 1c with a minimum *z*-threshold in both directions of 3.1.

#### Contrast maps for the hand and lips

To visualize the contrast maps for the hand and lip movements, estimates from the four finger-mapping scan runs for each session were averaged in a voxelwise manner using a fixed-effects model with a cluster-forming *z*-threshold of 3.1 and family-wise error-corrected cluster significance threshold of *P* < 0.05. Maps were then projected onto the participant’s cortical surface. These contrast maps (hand in red and lips in blue) are visualized in Fig. 1d with a minimum *z*-threshold of 33% the maximum participant-specific *z*-statistic.

For completion, the boundaries of the lip maps, for all participants who underwent an amputation across all sessions, are visualized in Fig. 3d. All maps were minimally thresholded at *z* > 4.5 to provide a complementary thresholding approach relative to Fig. 1d.

#### Hand topography across the 49 segments of the BA3b

Using the 49 segments of the BA3b (described above), we projected the neural activity for the hand (versus rest) for each hemisphere (contralateral to the hand being moved), session and participant. The average activity across all voxels in each segment was averaged to extract a single value per segment.

#### COG

To quantify changes in the hand, finger or lip topography, we computed the COG of activity (for a single body part) across the 49 BA3b segments. To do this, we first computed the weighted activity (*β*_*w*_) across the segments. To do this each segment number was multiplied by the average activity in the segment:

$${\beta }_{w}=\left(1\;x{\beta }_{1}\right)+\left(2x{\beta }_{2}\right)\ldots$$

To compute the COG, we then divided the sum of the weighted activity (*Σβ*_*w*_) by the sum of the activity (*Σ*β).

$${\mathrm{COG}}=\frac{\sum {\beta }_{w}}{\sum {{\beta }}}$$

When comparing changes in the COG for the hand or a finger, the COG for each post-session was subtracted from the average COG of the pre-sessions (for example, 3-month COG–pre. avg COG). A value greater than zero reflects the COG moving more medially in the post-session compared to the pre-session. A value less than zero reflects the post-session COG being more lateral compared to the pre-session COG.

#### Finger selectivity maps

To visualize the selectivity maps, estimates from the four finger-mapping scan runs for each session were averaged in a voxelwise manner using a fixed-effects model. When visualizing the clusters, we minimally thresholded each *z*-statistic at 33% the maximum *z*-statistic. We stacked the images such that the smallest cluster was the highest overlay (for example, the pinkie finger) and the largest cluster was the underlay. Finally, we applied a 70% opacity to the visualizations to capture multi-finger activity at each voxel.

#### Representative Ctrl participant body part maps

To provide an example visualization of the activity for each of the body parts shown in Fig. 3c, estimates from the four finger-mapping scan runs for each session were averaged in a voxelwise manner using a fixed-effects model, with a cluster-forming *z*-threshold of 3.1 and family-wise error-corrected cluster significance threshold of *P* < 0.05. We then visualized the *z*-statistic map for the contrast of lips > feet and all left fingers > feet on an inflated cortical surface and applied a threshold to each body part (*z* > 3.1).

#### Lip activity in the BA3b hand region

To test whether there was an increase in lip activity in the BA3b hand region, the average activity for all voxels (non-thresholded) in the ROI was computed for each session and each run. Activity was averaged across runs to compute a session estimate. When testing for a difference between the after and before amputation sessions, the activity for the two pre-amputation sessions was averaged for a pre-amputation average estimate. The activity in each post-amputation session (3 months, 6 months, 1.5/5 years) was then subtracted to the activity of the pre-amputation average.

### Winner-takes-all analysis

As a qualitative demonstration of our findings been compatible with previous studies investigating cortical reorganization that used a winner-takes-all approach, we applied a winner-takes-all analysis to S1 functional activity of the case study participants who underwent an amputation. Using each participant’s final post-amputation session data, we performed two variations of the analysis including the following conditions: (1) lips, hand and feet; or (2) lips and feet (excluding the hand). Each voxel was assigned exclusively to the condition with the highest activity. The resulting images were mapped to the participant’s cortical surface and are visualized in Extended Data Fig. 9.

### Multivoxel pattern analyses

We performed several multivoxel pattern analyses that can be broadly categorized into three themes: intra-finger; inter-finger; and inter-body part. In these measures, we were interested in capturing differences within a session and differences between sessions. For all these analyses, we only included voxels in the BA3b hand region.

#### Intra-finger

##### Pearson correlations

We first wanted to quantify changes in the pattern of activation for single fingers (intra-finger). We performed Pearson correlations on the beta weights for each finger using data from runs from different sessions (Fig. 2b and Extended Data Fig. 5). For between-session correlations, the beta weights (in our instance, contrast of parameter estimates) for each finger in the four scan runs were separated into partitions, each with two runs, and each set from different sessions. The activity in each two-run set was averaged at every voxel. A Pearson correlation was then performed between the averaged activity in each of the splits. We performed all unique two-run combinations between sessions (36 total combinations) and averaged these correlation coefficients to get a single value per finger. Between-session correlations were performed for all six unique session comparisons: Pre1 to Pre2, Pre1 to 3 months, Pre1 to 6 months, Pre2 to 3 months, Pre2 to 6 months and 3 months to 6 months. Additionally, for P1 and P2, correlations were performed for Pre1 to 1.5/5 years and Pre2 to 1.5/5 years. All correlation coefficients were then averaged and plotted in Extended Data Fig. 5. For a simpler visualization, we plotted just the first combination for each participant’s final scan relative to the pre-amputation average in Fig. 2b.

#### Inter-finger

We next wanted to quantify changes in the pattern of activation between finger pairs (inter-finger) using a decoding approach (Fig. 2d) and cross-validated Mahalanobis distances (Extended Data Fig. 6). Both approaches capture slightly different aspects of the representational structure^56^, which we elaborate on below.

For these two analyses, the beta weights from the first-level GLM for each participant were extracted and spatially pre-whitened using a multivariate noise normalization procedure (as described in ref. ^56^). This was done for each scan using the residuals from the GLM. We then used these noise-normalized beta weights for the next analyses.

##### Decoding

First, we performed a decoding analysis. A strength of this approach is that it provides an estimate for chance performance (50%), that is, it is a classification accuracy significantly greater than chance. For the case study participants who underwent an amputation, the decoding approach can tell us whether a decoder trained on pre-amputated finger pairs can correctly decode the same information on a phantom hand.

We used a linear SVM classifier (scikit-learn v.1.2.2; sklearn.svm, LinearSVC) to quantify the between-session decoding for each finger pair. Default parameters were used for the classifier. Classification accuracy above chance (50%) denotes that there is some amount of shared information between the training and testing datasets.

We trained the classifier on the noise-normalized beta weights for each finger pair (ten in total). The training and testing splits were performed using data from different sessions, such that the classifier was trained on each unique two-run combination from one session and tested on all unique two-run combinations in a separate session (36 combinations for each finger pair). We performed the same classification approach in the reverse direction (72 combinations in total) because the forward and reverse directions provide unique values. The accuracies for each finger pair for each two-run combination for each training and testing direction were then averaged. Between-session accuracies are shown in Fig. 1d.

##### Cross-validated Mahalanobis distances

Because our decoding analysis was performed at ceiling (close to 100%), we also performed a RSA using cross-validated Mahalanobis distances. The strength of this approach is that it computes a distance measure (continuous) rather than a binary decoding measure. As such, it is arguably more sensitive for capturing the inter-finger representational structure. Larger distances reflect more dissimilar (distinct) activity patterns and smaller distances reflect more similar patterns.

We performed this analysis using data from different sessions to compute between-session distances (our desired measure for representational stability over time). A distance cross-validated between sessions captures the stability of the information content.

We calculated the squared cross-validated Mahalanobis distance between activity patterns as:

$${d}^{\;2}\left({x}_{y},{x}_{z}\right)={\left({x}_{y}-{x}_{z}\right)}_{A}^{T}{\sum }^{-1}{\left({x}_{y}-{x}_{z}\right)}_{B}$$

where ${\left({x}_{y}-{x}_{z}\right)}_{A}$ corresponds to the difference between the activity patterns of conditions *y* (for example, thumb) and *z* (for example, index finger) in partition A, and *Σ* refers to the voxelwise noise covariance matrix. We performed this procedure over all possible two-run cross-validation folds and then averaged the resulting distances across folds. There were 36 unique cross-validation folds between sessions. Note that the cross-validated distance gives you the same distance value regardless of whether it is assigned partition A or partition B. Between-session distances are shown in Extended Data Fig. 6.

##### Typicality

To quantify a measure that represents the degree of ‘normality’ of the hand representation, we computed a representational typicality measure^10^. For each participant’s nondominant left hand, we extracted the ten crossnobis distances for the Pre-3 month and Pre-6 month comparisons. We then averaged these vectors across all able-bodied participants to get an average typical hand pattern. We then performed a Spearman rho correlation between the cross-validated Mahalanobis finger-pair distances for each participant’s affected or nondominant (left) hand and the average typical hand pattern. When comparing a Ctrl participant to the Ctrl mean, the respective participant was left out from the estimation of the Ctrl mean distances. These values are depicted in Extended Data Fig. 6.

#### Inter-body part

Finally, we wanted to quantify changes in the pattern of activation between the thumb, lips and feet in the S1 hand region. We computed the cross-validated Mahalanobis distances between these body parts in the same manner as the inter-finger analysis. The thumb to lips distances are plotted Fig. 3. The distances between all conditions are plotted in Extended Data Fig. 7.

### Statistical analyses

All statistical analyses were performed either with Python scripts using scipy.stats and statsmodels.stats.multitest or JASP (v.0.17.2.1). Normality was ascertained using a Shapiro–Wilk test. For most of the analyses, to test whether a case study participant was significantly different from the Ctrl group, we used Crawford & Howell’s method, which provides a point estimate of the abnormality of the distance of each case from a Ctrl sample^57^. For all Crawford tests, we report uncorrected, two-tailed *P* values. When comparing estimates to zero or chance decoding (50%), we used a two-tailed, one-sample *t*-test. When testing for a decrease in measures within-participant, we used a Wilcoxon signed-rank test. When further testing for differences between hands within-participant, we performed a Wilcoxon signed-rank test on the classification accuracy values and a paired samples *t*-test on the Mahalanobis distances. The resulting *P* values were *z*-transformed and are plotted in Supplementary Fig. 1. Additionally for the correlation analyses, Pearson correlations were used for the intra-finger multivoxel pattern analysis and Spearman correlations were used for the typicality analysis.

Across all our previous studies, we operationally defined amputees’ intact hand as their de facto dominant hand, and as such have always compared the nondominant hand of Ctrls to the missing hand of amputees (for example, see refs. ^9,14,37,41,58–60^). Therefore, across all case study to Ctrl comparison analyses, we statistically compared (and plotted) the left (nondominant) hand side of Ctrls to the case study participants missing hand side.

### Reporting summary

Further information on research design is available in the Nature Portfolio Reporting Summary linked to this article.

## Online content

Any methods, additional references, Nature Portfolio reporting summaries, source data, extended data, supplementary information, acknowledgements, peer review information; details of author contributions and competing interests; and statements of data and code availability are available at 10.1038/s41593-025-02037-7.

## Supplementary information

Supplementary Information Supplementary Table 1, Figs. 1–4 and Methods.

Reporting Summary

Supplementary Video 1 Video of the case study participants before and after arm amputation.

Supplementary Video 2 Video of the case study participants moving the affected fingers before amputation and the phantom fingers after amputation.

## Figures and Tables

**Extended Data Table 1:**
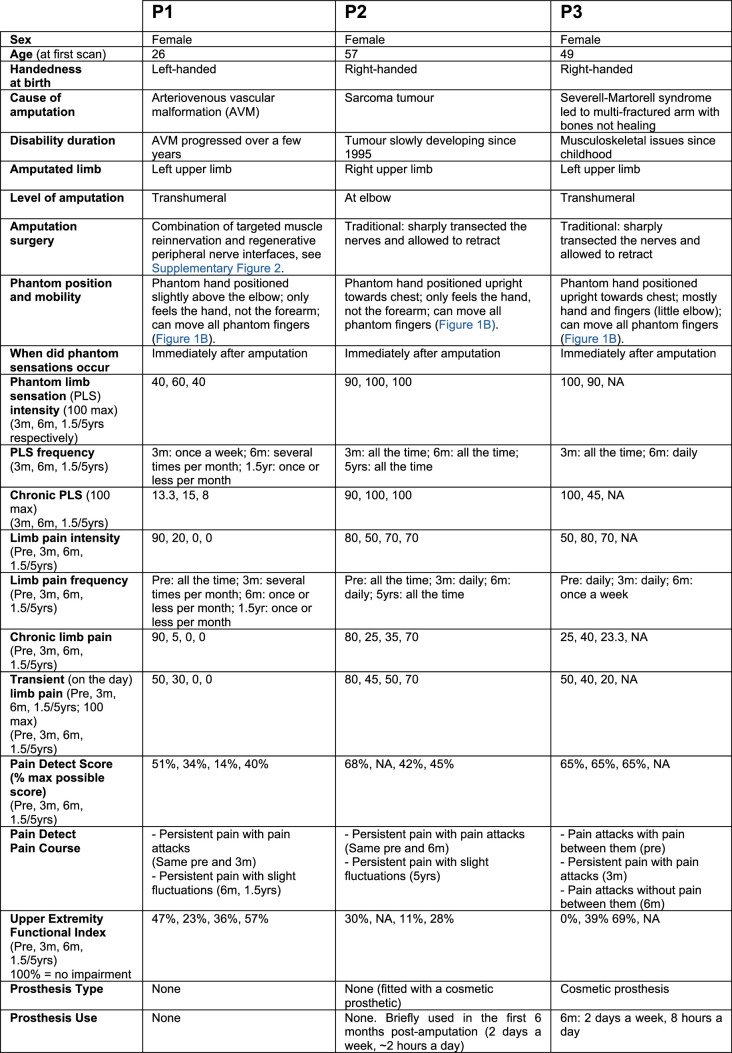
Demographics of the case study participants who underwent an amputation

PLS = phantom limb sensation; Limb pain reflects pre-amputation limb pain or post-amputation phantom limb pain. Frequency scores: 1 – all the time, 2 – daily, 3 – weekly, 4 – several times per month, and 5 – once or less per month. Chronic pain/sensation values were calculated by dividing intensity by frequency. NA = not available/applicable. Upper extremity functional index measures participant difficulty with performing activities due to their missing limb.

## Data Availability

Data for the primary results have been made publicly available (https://osf.io/s9hc2/).

## Code availability

The code for the primary results has been made publicly available (https://github.com/hunterschone/longitudinal-amputation).
