# Supplementary material for: Stable cortical body maps before and after arm amputation
Source: Nat Neurosci. 2025 Aug 21;28(10):2015–21. doi: 10.1038/s41593-025-02037-7 (PMC12477700; doi:10.1038/s41593-025-02037-7)
Supplement: Supplementary file 1 — Supplementary Table 1, Figs. 1–4 and Methods. [file 41593_2025_2037_MOESM1_ESM.pdf]

nature neuroscience

Supplementary information https://doi.org/10.1038/s41593-025-02037-7

Stable cortical body maps before and after arm amputation

In the format provided by the authors and unedited

Supplementary Information

Inventory

Supplementary Display Items

- Supplementary Table 1. *Pearson correlations for controls finger representations across sessions.*
- Supplementary Figure 1. *Comparing finger selective information of the affected hand versus the unaffected hand.*
- Supplementary Figure 2. *Summary of P1's amputation procedure.*
- Supplementary Figure 3. *Comparing case study P1's hand and lip activity to 2 longitudinal able-bodied participant datasets: older and younger controls.*
- Supplementary Figure 4. *Intact finger kinematics during mirrored and phantom finger movements.*

Supplementary Methods

Supplementary Display Items

**Session Comparison**	**Hemisphere/Hand**	**Finger**	**Correlation Coefficient** (r) (mean ± std)
Pre to 3m	R/L	D1	0.83 ± 0.08
Pre to 3m	R/L	D2	0.84 ± 0.10
Pre to 3m	R/L	D3	0.88 ± 0.06
Pre to 3m	R/L	D4	0.90 ± 0.04
Pre to 3m	R/L	D5	0.89 ± 0.04
Pre to 6m	R/L	D1	0.85 ± 0.07
Pre to 6m	R/L	D2	0.84 ± 0.07
Pre to 6m	R/L	D3	0.89 ± 0.05
Pre to 6m	R/L	D4	0.90 ± 0.04
Pre to 6m	R/L	D5	0.89 ± 0.04
Pre to 3m	L/R	D1	0.80 ± 0.12
Pre to 3m	L/R	D2	0.79 ± 0.13
Pre to 3m	L/R	D3	0.83 ± 0.12
Pre to 3m	L/R	D4	0.87 ± 0.07
Pre to 3m	L/R	D5	0.88 ± 0.07
Pre to 6m	L/R	D1	0.78 ± 0.16
Pre to 6m	L/R	D2	0.79 ± 0.13
Pre to 6m	L/R	D3	0.84 ± 0.10
Pre to 6m	L/R	D4	0.87 ± 0.08
Pre to 6m	L/R	D5	0.87 ± 0.07

Supplementary Table 1. Pearson correlations for controls finger representations across sessions.

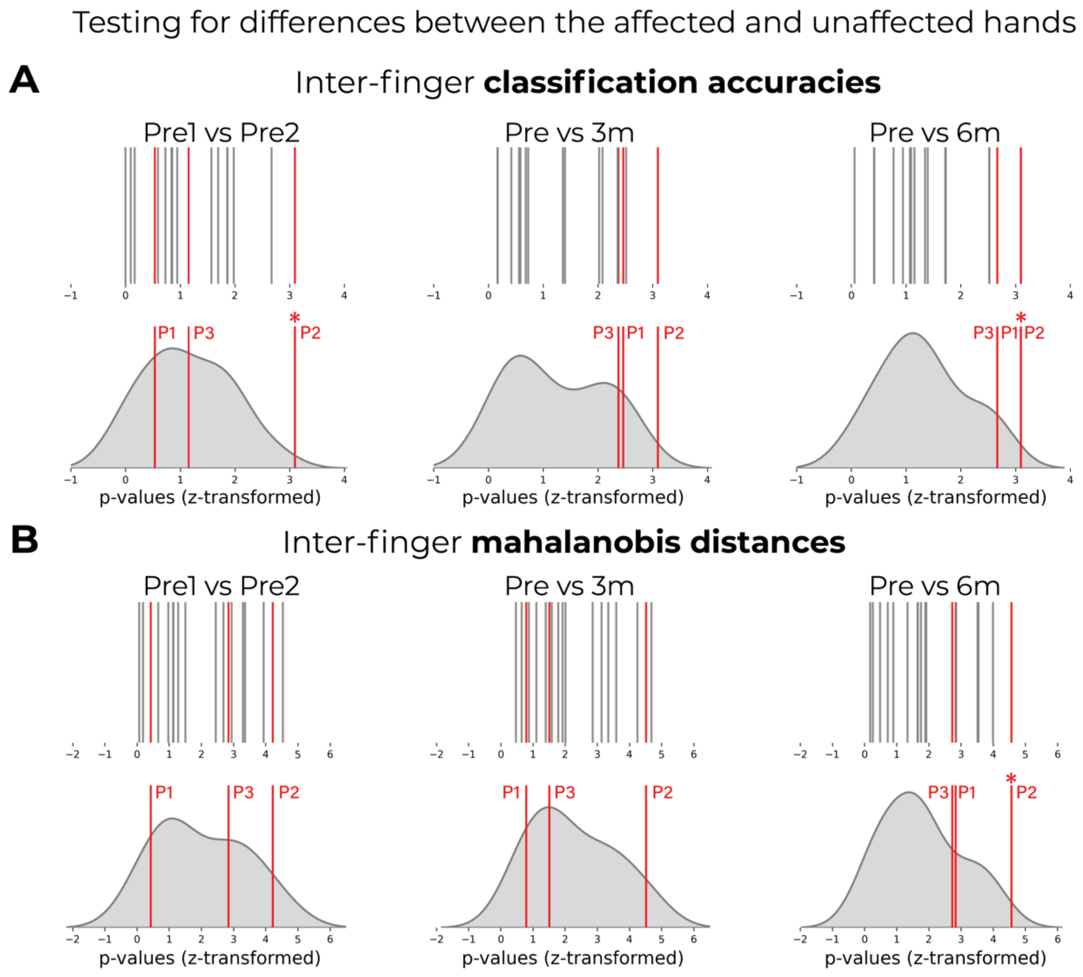

Supplementary Figure 1. Comparing finger selective information of the affected hand versus the unaffected hand. *Using the inter-finger classification accuracy values depicted in Figure 2D and Supp. Figure 6B, we performed paired statistical tests comparing values between hands, within-participant.* ***(A)*** *For the classification accuracy values, we performed paired Wilcoxon Signed-Ranks tests and z-transformed the resulting p-values (plotted in panel A). We then performed the Crawford t-tests on the z-transformed p-values. We observed 2 of 3 participants (P1 and P2) exhibited significantly decreased finger selective information for the affected hand than the unaffected hand, at the 6-month timepoint, relative to able-bodied controls (3 Crawford t-tests per participant; Pre1-Pre2: P1: p_{uncorr}=0.45; P2: p_{uncorr}=0.02; P3: p_{uncorr}=0.97; Pre-3m: P1: p_{uncorr}=0.18; P2: p_{uncorr}=0.05; P3: p_{uncorr}=0.21; Pre-6m: P1: p_{uncorr}=0.03; P2: p_{uncorr}=0.03; P3: p_{uncorr}=0.10). Though, it's worth noting P2 also exhibited decreased inter-finger classification accuracies for the affected hand than the unaffected hand at baseline (pre-amputation), relative to controls.* ***(B)*** *For the Mahalanobis distance values, we performed paired samples t-tests and z-transformed the resulting p-values (plotted in panel B). We observed only P2 exhibited significantly decreased finger selective information for the affected*

hand than the unaffected hand, at the 6-month timepoint, relative to able-bodied controls (3 Crawford t-tests per participant; Pre1-Pre2: P1: p_{uncorr}=0.27; P2: p_{uncorr}=0.13; P3: p_{uncorr}=0.56; Pre-3m: P1: p_{uncorr}=0.29; P2: p_{uncorr}=0.10; P3: p_{uncorr}=0.58; Pre-6m: P1: p_{uncorr}=0.40; P2: p_{uncorr}=0.03; P3: p_{uncorr}=0.44). The grey values depict the able-bodied control participants. The top rows of each panel depict the individual participant's as vertical lines. The bottom rows of each panel depict a distribution of the control participants (using a probability kernel density estimation function). The individual red lines depict the values for each of our case-study participants.

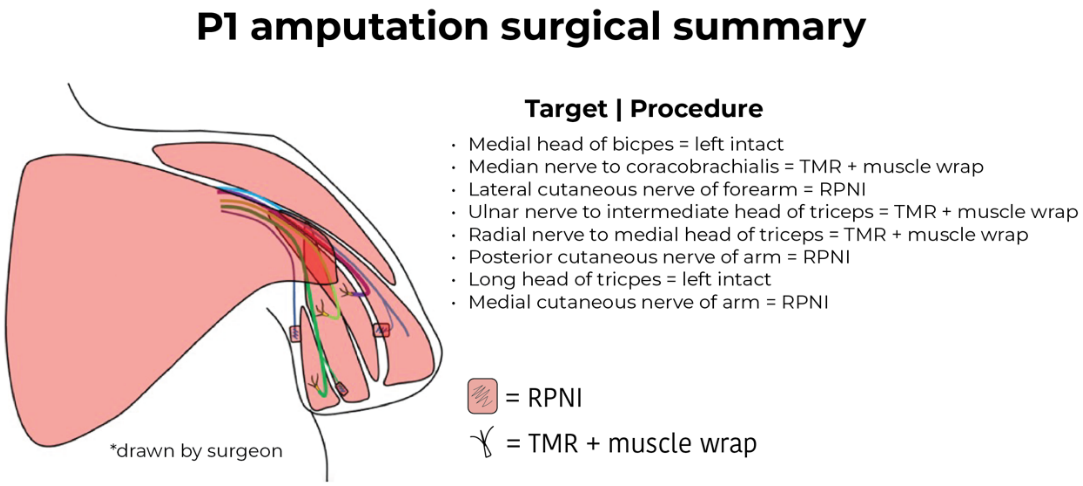

Supplementary Figure 2. Summary of P1's amputation procedure. *An illustration depicting the unique amputation surgery of P1's left arm, as well as summary of the procedures performed for each respective nerve. TMR=targeted muscle reinnervation; RPNI=regenerative peripheral nerve.*

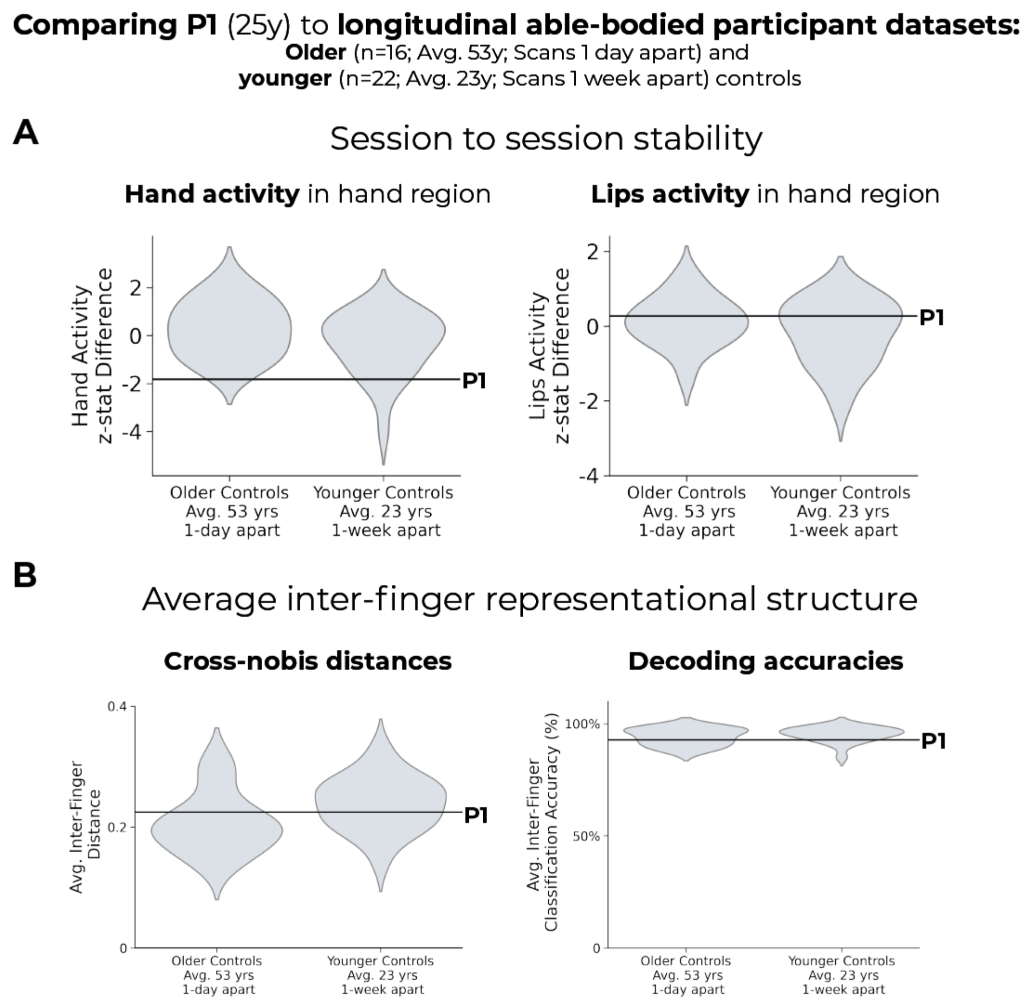

Supplementary Figure 3. Comparing case study P1's hand and lip activity to 2 longitudinal able-bodied participant datasets: older and younger controls. *As the longitudinal able-bodied controls were age-matched to P2 and P3, we investigated whether younger able-bodied controls (see Methods) showed significant differences on multiple measures compared to the older controls. The older controls are the longitudinal controls described in the main text. The younger controls performed the same task on the same scanner, 2 scans separated by 1-week.* ***.*** *We examined the session-to-session stability in our primary univariate and multivariate measures. First, for the univariate measures, younger and older controls showed similar session to session differences in* ***(A)*** *hand activity in the S1 hand region (independent samples t-test: t(37)=1.7, p=0.09), and* ***(B)*** *lip activity in the hand region (t(37)=1.3, p=0.18). Further, P1's pre-amputation scan data (black line) showed no significant difference between older controls and younger controls for either body-part (hand: P1 vs. Older: t(15)=-1.7, p=0.09; P1 vs. Younger: t(21)=-1.1, p=0.2; lips: P1 vs. Older: t(15)=0.2, p=0.8; P1 vs. Younger: t(21)=0.52, p=0.6). Grey violin plots reflect controls data (95% percentile interval).* ***(C)*** *Next, for the multivariate measures, younger controls showed a trend for higher average inter-finger representational*

structure compared to older controls, in the cross-nobis distances (t(37)=-1.95, p=0.06), but not the decoding (t(37)=-0.87, p=0.38). P1's pre-amputation session data was not different than the older or younger control groups for either measure (cross-nobis: P1 vs. Older: t(15)=0.32, p=0.75; P1 vs. Younger: t(21)=-0.32, p=0.74; decoding: P1 vs. Older: t(15)=-0.35, p=0.72; P1 vs. Younger: t(21)=-0.70, p=0.48). All other annotations are the same as those described in Figure 2.

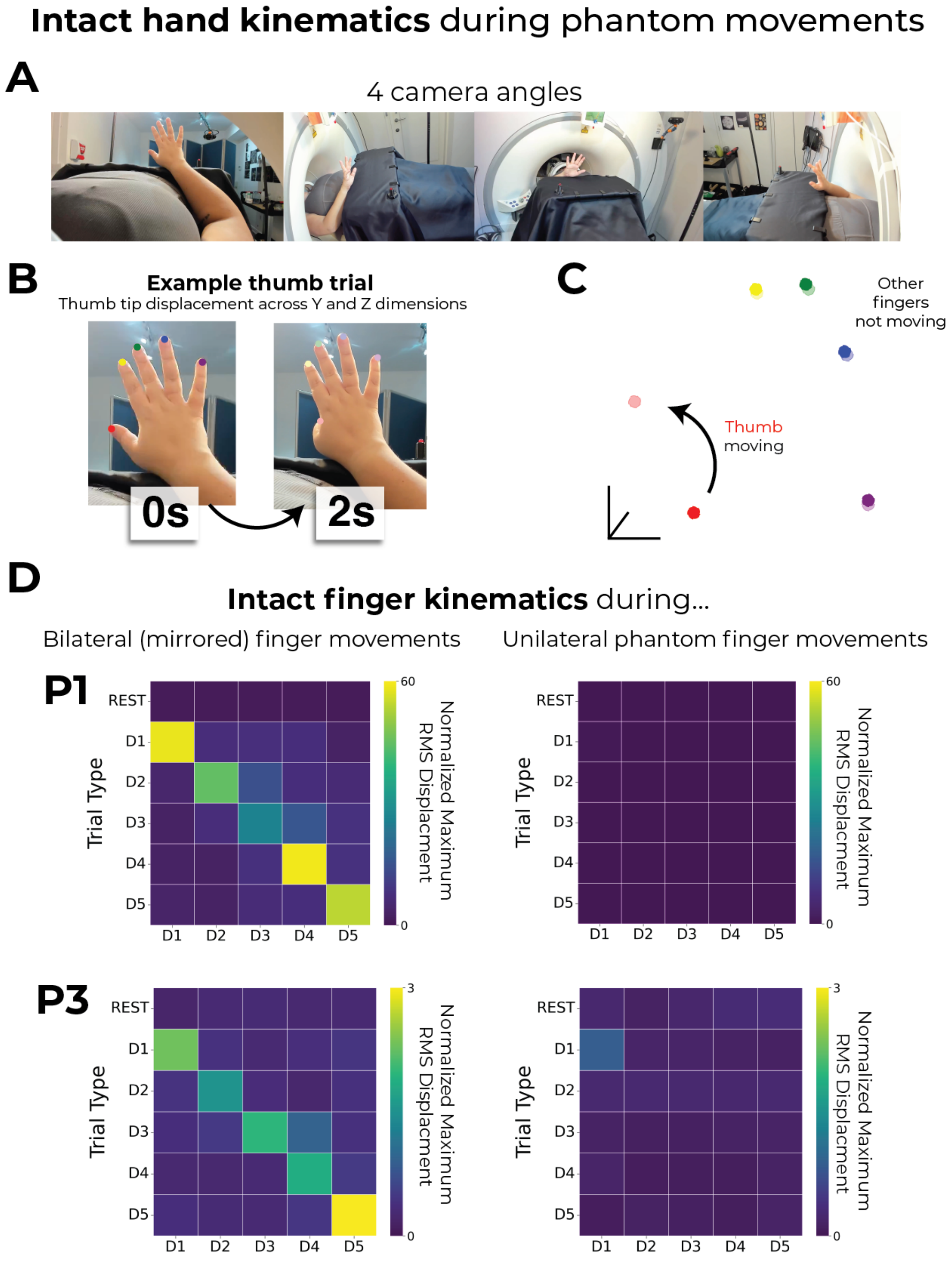

Supplementary Figure 4. Intact finger kinematics during mirrored and phantom finger movements. (A) *To test whether the intact fingers are being moved simultaneously during phantom finger movements, we tested 2 of the 3 case-study participants on a finger tapping task. Each participant was positioned inside an MRI scanner. We visually cued each participant to perform a finger flexion movement (each 2-seconds; 5 fingers or REST; 7 repetitions per condition). There were two blocks: bilateral (mirrored) finger movements, where participants were told to mirror the movements of the intact and phantom fingers,*

and unilateral phantom finger movements, where participants were told to move the phantom fingers. Participants were randomly cued which finger to move (or REST). We recorded kinematics of the intact fingers, using 4 cameras (Logitech brio, 1080p, 60fps). ***(B-C)*** *Using Anipose's triangulation function*[32] *to triangulate the 4 cameras into 3D coordinates, we defined the 3D coordinates of the tip of each finger. Using the 3D coordinates, we then computed the root mean square (RMS) displacement of each dimension (x, y, z) within a trial. Across dimensions, we selected the dimension with the highest RMS displacement. We then averaged across repetitions of the same trial type. Finally, we normalize these values relative to the RMS displacement observed in the REST condition, effectively capturing relative movement magnitude. We provide a single trial visualization of each finger's 3D coordinates (for the y and z dimensions) at the first (dark colours) and last (light colours) timepoints of a single move thumb trial. Note the distinct individuation of the thumb and not the other fingers.* ***(D)*** *We observed that while bilateral mirror finger movements show clear finger individuation of the intact fingers (plots on the left), the intact fingers do not move during phantom finger movements (plots on the right).*

Supplementary Methods

Participants

We pooled two previously collected cross-sectional task fMRI datasets[1] which included: (1) a group of chronic amputees (n=26) and (2) a secondary group of able-bodied controls (n=18). The chronic amputee group included 26 upper-limb amputee participants [4 females; mean age ± std = 51.1 ± 10.6; 13 missing left upper-limb; level of amputation: 17 transradial, 8 transhumeral and 1 at wrist; mean years since amputation ± std = 23.5 ± 13.5]. The secondary able-bodied control group included 18 able-bodied participants [7 females; mean age ± std = 43.1 ± 14.62; 11 right-handed]. For complete demographics, see Supplementary Table 2 below.

Subj	Group	Dataset	Age	Gender	Missing Side	Level of amputation	Years since amputation
A1	Amputee	1	58	Male	Left	Transradial	14
A2	Amputee	1	46	Female	Left	Transradial	16
A3	Amputee	1	50	Female	Left	Transradial	3
A4	Amputee	1	53	Male	Left	Transhumeral	34
A5	Amputee	1	42	Male	Right	Transradial	18
A6	Amputee	1	61	Male	Right	Transradial	21
A7	Amputee	1	60	Male	Right	Transradial	42
A8	Amputee	1	47	Male	Right	Transhumeral	21
A9	Amputee	1	68	Male	Left	Transradial	12
A10	Amputee	1	49	Male	Right	Transradial	5
A11	Amputee	1	57	Male	Left	Transradial	29
A12	Amputee	1	53	Male	Left	Transradial	33
A13	Amputee	1	28	Female	Right	Transradial	10

A14	Amputee	1	29	Male	Left	Transradial	11
A15	Amputee	1	43	Male	Right	Transradial	20
A16	Amputee	1	55	Male	Left	Transradial	12
A17	Amputee	1	40	Female	Right	Transhumeral	9
A18	Amputee	1	46	Male	Right	Transradial	33
A19	Amputee	2	38	Male	Left	Transhumeral	27
A20	Amputee	2	49	Male	Left	Transhumeral	16
A21	Amputee	2	69	Male	Right	Transhumeral	52
A22	Amputee	2	62	Male	Left	At wrist	31
A23	Amputee	2	56	Male	Left	Transradial	54
A24	Amputee	2	55	Male	Right	Transhumeral	26
A25	Amputee	2	50	Male	Right	Transhumeral	23
A26	Amputee	2	66	Male	Right	Transradial	40
C1	Control	1	28	Female	NA	NA	NA
C2	Control	1	61	Female	NA	NA	NA
C3	Control	1	18	Female	NA	NA	NA
C4	Control	1	51	Female	NA	NA	NA
C5	Control	1	33	Male	NA	NA	NA
C6	Control	1	54	Male	NA	NA	NA
C7	Control	2	29	Male	NA	NA	NA
C8	Control	2	24	Female	NA	NA	NA
C9	Control	2	52	Male	NA	NA	NA
C10	Control	2	47	Male	NA	NA	NA
C11	Control	2	39	Female	NA	NA	NA
C12	Control	2	32	Male	NA	NA	NA
C13	Control	2	53	Male	NA	NA	NA
C14	Control	2	38	Male	NA	NA	NA
C15	Control	2	67	Female	NA	NA	NA
C16	Control	2	41	Male	NA	NA	NA
C17	Control	2	41	Male	NA	NA	NA
C18	Control	2	69	Male	NA	NA	NA

Supplementary Table 2. Demographics of cross-sectional datasets. Previous dataset denotes which dataset each subject came from.

For both datasets, informed consent and consent to publish was obtained in accordance with ethical standards set out by the Declaration of Helsinki. All participants gave full written informed consent for their participation, data storage and dissemination. For dataset 1, ethical approval was granted by the NHS National Research Ethics Committee (18/LO/0474) and University College London's research ethics committee (9937/001). The experimental procedures for this dataset have not been previously published, so they will be described below. For dataset 2, ethical approval was granted by the University of Oxford's Medical Sciences inter-divisional research ethics committee (MS-IDREC-C2-2015-012). The experimental procedures for this dataset have been previously described (see ref. [1]; the full study protocol can also be found at

https://osf.io/gmvua/). For this dataset, I will focus solely on the fMRI task and functional data analyses used in the present manuscript.

Scanning Procedures
Across both datasets, each participants MRI session consisted of a structural scan and a body localizer scan, which we report below. While additional functional tasks were performed, they will not be reported here.

fMRI Task Design
Body localizer scan
During a single body localizer scan, participants were visually cued to move each hand, each elbow, purse their lips and curl their toes. The different movement conditions and a rest (fixation) cue were presented in 12-second blocks and repeated 4 times.

MRI Data Acquisition
Dataset 1
The MRI measurements were obtained using a 3-Tesla Prisma scanner (Siemens, Erlangen, Germany) with a 32-channel head coil. Anatomical data were acquired using a T1-weighted magnetization prepared rapid acquisition gradient echo sequence (MPRAGE) with the parameters: TR = 2.53 s, TE = 3.34 ms, FOV = 256 mm, flip angle = 7˚, and voxel size = 1 mm isotropic resolution. Functional data based on the blood oxygenation level-dependent signal were acquired using a multiband gradient echo-planar T2*-weighted pulse sequence[2] with the parameters: TR = 1.45 s, TE = 35 ms, flip-angle = 70˚, multi-band acceleration factor = 4, FOV = 212 mm, matrix size of 106 x 106, and voxel size = 2 mm isotropic resolution. Seventy-two slices, with a slice thickness of 2 mm and no slice gap, were oriented in the anterior commissure – posterior commissure, covering the whole cortex, with partial coverage of the cerebellum. The single functional run of the body localizer task consisted of 414 volumes.

Dataset 2
MRI data acquisition parameters for this dataset have been previously reported in detail (see [1]). Like dataset 1, the single functional run also consisted of 414 volumes.

fMRI Analysis
Functional MRI data for both datasets was processed similarly to the longitudinal functional data. Functional MRI data processing was carried out using FMRIB's Expert Analysis Tool (FEAT; Version 6.0), part of FSL (FMRIB's Software Library, www.fmrib.ox.ac.uk/fsl) and Connectome Workbench (humanconnectome.org) software, in combination with Matlab scripts (R2023b, Mathworks Inc, Natick, MA). Registration of the functional data to the high-resolution structural image was carried out using the boundary-based registration algorithm [3]. Registration of the high resolution structural to standard space images was carried out using FLIRT [4,5] and was then further refined using FNIRT nonlinear registration [6,7]. The following pre-statistics processing was applied; motion correction using MCFLIRT [5]; non-brain removal using

BET[8]; spatial smoothing using a Gaussian kernel of FWHM 3mm; grand-mean intensity normalisation of the entire 4D dataset by a single multiplicative factor; high-pass temporal filtering (Gaussian-weighted least-squares straight line fitting, with sigma=50s).

We applied a general linear model (GLM) as implemented in FEAT, to each participant's functional run. Time-series statistical analysis was carried out using FILM with local autocorrelation correction[9]. The time series model included trial onsets convolved with a double gamma HRF function, six motion parameters were added as confound regressors. Each movement condition was modelled separately. For each participant, parameter estimates of the each of the different conditions (versus rest) and GLM residuals of all voxels were extracted from this first-level analysis. All subsequent analyses were performed with the functional data aligned to the standard space.

We next visualized the hand and lip cortical maps for each individual subject (Extended Data Figure 8) and at the group-level (Figure 3F). First, to visualize the maps for the hand and lips, estimates from the four finger-mapping scan runs were averaged in a voxel wise manner using a fixed effects model with a cluster forming z-threshold of 3.1 and family-wise error corrected cluster significance threshold of $p < 0.05$. To visualize all data on the same hemisphere, for participants missing their left arm (or controls with a non-dominant left arm), we flipped the hemisphere side, such the affected or non-dominant hemisphere for all participants was the left hemisphere. Maps were then projected onto a standard cortical surface using workbench command's volume-to-surface-mapping function which included a ribbon constrained mapping method. These contrast maps (hand in red and lips in blue) are visualized in Extended Data Figure 8 with a minimum z-threshold of 50% the maximum subject-specific z-statistic.

Next, we visualized the group-level amputee maps for the hand and lips. Using the hemisphere flipped images described above, group level analysis of maps was carried out using FMRIB's Local Analysis of Mixed Effects (FLAME) with a cluster forming z-threshold of 3.1 and family-wise error corrected cluster significance threshold of $p < 0.05$. The group-level activity map was mapped to the standard cortical surface and applied with an opaque filter to any activity beyond the S1 ROI (Figure 3).

Finally, we provided a line plot visualizing hand and lip topography across BA3b. Using the 49 segments of BA3b, we projected the (hemisphere flipped) cortical activity for the hand and lips (versus rest) for each participant. The average activity across all voxels within each segment was averaged to extract a single value per segment. The group mean and standard error of the amputee participants was plotted for each segment in Figure 3.

Regions of Interest
Two regions of interest (ROI) were used with these datasets: (1) 49 segments of BA3b and (2) S1 hand region of interest. These ROIs were defined in the same way as described in the manuscript's Methods section. The sole exception being that these

ROIs were kept in standard space when analyzing the functional data of the cross-sectional datasets.

References

1. Tucciarelli, R. *et al.* Does Ipsilateral Remapping Following Hand Loss Impact Motor Control of the Intact Hand? *J. Neurosci.* **44**, (2024).
2. Uğurbil, K. *et al.* Pushing spatial and temporal resolution for functional and diffusion MRI in the Human Connectome Project. *NeuroImage* **80**, 80–104 (2013).
3. Greve, D. N. & Fischl, B. Accurate and robust brain image alignment using boundary-based registration. *NeuroImage* **48**, 63–72 (2009).
4. Jenkinson, M. & Smith, S. A global optimisation method for robust affine registration of brain images. *Medical Image Analysis* **5**, 143–156 (2001).
5. Jenkinson, M., Bannister, P., Brady, M. & Smith, S. Improved Optimization for the Robust and Accurate Linear Registration and Motion Correction of Brain Images. *NeuroImage* **17**, 825–841 (2002).
6. Andersson, J. L. R., Jenkinson, M. & Smith, S. M. Non-linear optimisation. FMRIB technical report TR07JA1. *FMRIB Technical Report* 16 (2007) doi:10.1109/EMOBILITY.2010.5668100.
7. Andersson, J. L. R., Jenkinson, M. & Smith, S. Non-linear registration, aka spatial normalization. *FMRIB Technical Report* (2007).
8. Smith, S. M. Fast robust automated brain extraction. *Human Brain Mapping* **17**, 143–155 (2002).
9. Woolrich, M. W., Ripley, B. D., Brady, M. & Smith, S. M. Temporal Autocorrelation in Univariate Linear Modeling of FMRI Data. *NeuroImage* **14**, 1370–1386 (2001).
