# Supplementary material for: Stable cortical body maps before and after arm amputation
Source: Nat Neurosci. 2025 Aug 21;28(10):2015–21. doi: 10.1038/s41593-025-02037-7 (PMC12477700; doi:10.1038/s41593-025-02037-7)
Supplement: Supplementary file 2 — Reporting Summary [file 41593_2025_2037_MOESM2_ESM.pdf]

Corresponding author(s): Hunter R. Schone
Last updated by author(s): 02/20/2025

# Reporting Summary

Nature Portfolio wishes to improve the reproducibility of the work that we publish. This form provides structure for consistency and transparency in reporting. For further information on Nature Portfolio policies, see our Editorial Policies and the Editorial Policy Checklist.

## Statistics

For all statistical analyses, confirm that the following items are present in the figure legend, table legend, main text, or Methods section.

| n/a | Confirmed | |
| --- | --- | --- |
| ☐ | ☒ | The exact sample size (*n*) for each experimental group/condition, given as a discrete number and unit of measurement |
| ☐ | ☒ | A statement on whether measurements were taken from distinct samples or whether the same sample was measured repeatedly |
| ☐ | ☒ | The statistical test(s) used AND whether they are one- or two-sided<br>*Only common tests should be described solely by name; describe more complex techniques in the Methods section.* |
| ☐ | ☒ | A description of all covariates tested |
| ☐ | ☒ | A description of any assumptions or corrections, such as tests of normality and adjustment for multiple comparisons |
| ☐ | ☒ | A full description of the statistical parameters including central tendency (e.g. means) or other basic estimates (e.g. regression coefficient) AND variation (e.g. standard deviation) or associated estimates of uncertainty (e.g. confidence intervals) |
| ☐ | ☒ | For null hypothesis testing, the test statistic (e.g. *F*, *t*, *r*) with confidence intervals, effect sizes, degrees of freedom and *P* value noted<br>*Give P values as exact values whenever suitable.* |
| ☐ | ☒ | For Bayesian analysis, information on the choice of priors and Markov chain Monte Carlo settings |
| ☐ | ☒ | For hierarchical and complex designs, identification of the appropriate level for tests and full reporting of outcomes |
| ☐ | ☒ | Estimates of effect sizes (e.g. Cohen's *d*, Pearson's *r*), indicating how they were calculated |

*Our web collection on statistics for biologists contains articles on many of the points above.*

## Software and code

Policy information about availability of computer code

| | |
| --- | --- |
| Data collection | Presentation software included PsychoPy (v2021.1.1). |
| Data analysis | Imaging software included FMRIB'S FEAT (v6), part of FSL, and Freesurfer (v7.1.1). All statistical analyses were performed using JASP (v0.17.21). All data was analyzed using custom Python (version 3) scripts. Code used in the study can be accessed at https://github.com/hunterschone/longitudinal-amputation. |

For manuscripts utilizing custom algorithms or software that are central to the research but not yet described in published literature, software must be made available to editors and reviewers. We strongly encourage code deposition in a community repository (e.g. GitHub). See the Nature Portfolio guidelines for submitting code & software for further information.

## Data

Policy information about availability of data

All manuscripts must include a data availability statement. This statement should provide the following information, where applicable:

- Accession codes, unique identifiers, or web links for publicly available datasets
- A description of any restrictions on data availability
- For clinical datasets or third party data, please ensure that the statement adheres to our policy

Data for the primary results and supplementary methods have been made publicly available (https://osf.io/s9hc2/).

## Research involving human participants, their data, or biological material

Policy information about studies with human participants or human data. See also policy information about sex, gender (identity/presentation), and sexual orientation and race, ethnicity and racism.

| | |
|---|---|
| Reporting on sex and gender | The participants who underwent planned hand amputations included 3 volunteers: P1 [female; age = 26; lefthanded; left transhumeral amputation], P2 [female; age = 57; left-handed; right at elbow amputation], P3 [female; age = 49; right-handed; left transhumeral amputation], were recruited through the National Health Service. The longitudinal able-bodied control group included 16 able-bodied volunteers [9 females; mean age ± std = 53.1 ± 6.37; all right-handed]. The chronic amputee group included 26 upper-limb amputee volunteers [4 females; mean age ± std = 51.1 ± 10.6; 13 missing left upper-limb; level of amputation: 17 transradial, 8 transhumeral and 1 at wrist; mean years since amputation ± std = 23.5 ± 13.5], which were recruited through the NHS. The secondary able-bodied control group included 18 able-bodied volunteers [7 females; mean age ± std = 43.1 ± 14.62; 11 right-handed]. Information on sex was self-reported by the volunteers. All able-bodied participants were recruited through University College London and the London metro area. |
| Reporting on race, ethnicity, or other socially relevant groupings | Not applicable |
| Population characteristics | See above. |
| Recruitment | All amputee participants were recruited via NHS participant identification centres. There were no self-selection biases that would impact our results. All able-bodied participants were recruited through University College London and the London metro area. |
| Ethics oversight | The study and its experimental procedures were approved by the NHS National Research Ethics Committee (18/LO/0474). |

Note that full information on the approval of the study protocol must also be provided in the manuscript.

# Field-specific reporting

Please select the one below that is the best fit for your research. If you are not sure, read the appropriate sections before making your selection.

☐ Life sciences ☒ Behavioural & social sciences ☐ Ecological, evolutionary & environmental sciences

For a reference copy of the document with all sections, see nature.com/documents/nr-reporting-summary-flat.pdf

# Behavioural & social sciences study design

All studies must disclose on these points even when the disclosure is negative.

| | |
|---|---|
| Study description | Quantitative experimental |
| Research sample | "The participants who underwent planned hand amputations included 3 volunteers: P1 [female; age = 26; left-handed; left transhumeral amputation], P2 [female; age = 57; left-handed; right at elbow amputation], P3 [female; age = 49; right-handed; left transhumeral amputation], were recruited through the National Health Service. The longitudinal able-bodied control group included 16 able-bodied volunteers [9 females; mean age ± std = 53.1 ± 6.37; all right-handed]. The chronic amputee group included 26 upper-limb amputee volunteers [4 females; mean age ± std = 51.1 ± 10.6; 13 missing left upper-limb; level of amputation: 17 transradial, 8 transhumeral and 1 at wrist; mean years since amputation ± std = 23.5 ± 13.5], which were recruited through the NHS. The secondary able-bodied control group included 18 able-bodied volunteers [7 females; mean age ± std = 43.1 ± 14.62; 11 right-handed]. Due to the rarity of identifying and testing participants pre-amputation, the sample size was based on the total number of amputees that could be successfully recruited. The researcher was not blinded to experimental condition and/or the study hypothesis." |
| Sampling strategy | Due to the rarity of identifying and testing participants pre-amputation, the sample size was based on the total number of amputees that could be successfully recruited. |
| Data collection | "There 3 data-types reported in the study: (1) fMRI data, (2) kinematic data and (3) questionnaire data. MRI images were obtained using a 3-Tesla Prisma scanner (Siemens, Erlangen, Germany) with a 32-channel head coil. Kinematic data was acquired by video recordings using 4 Logitech brio cameras. Questionnaire data was acquired via paper and pen. For all sessions, a single researcher and the research participant were present." |
| Timing | All data collection took place between May 4, 2019 to May 17, 2024. |
| Data exclusions | No data were excluded. |
| Non-participation | Over a 7-year period and across multiple NHS sites in the UK, we recruited 18 potentil patients preparing to undergo hand amputations. Due to a multitude of factors (e.g., MRI safety contraindications, no hand motor control, age outside ethics, high level |

of disability), we could only perform pre-amputation testing on 6 patients. Due to additional factors (complications during surgery, general health, retractions) we successfully completed our full testing procedure on 3 patients. For the able-bodied controls, 4 volunteers did not complete their testing, due to drop-out and incidental findings captured in the MRI sessions.

Randomization

No randomization was performed because all participants underwent the same testing procedures.

# Reporting for specific materials, systems and methods

We require information from authors about some types of materials, experimental systems and methods used in many studies. Here, indicate whether each material, system or method listed is relevant to your study. If you are not sure if a list item applies to your research, read the appropriate section before selecting a response.

## Materials & experimental systems

| n/a | Involved in the study |
|---|---|
| ☒ | ☐ Antibodies |
| ☒ | ☐ Eukaryotic cell lines |
| ☒ | ☐ Palaeontology and archaeology |
| ☒ | ☐ Animals and other organisms |
| ☒ | ☐ Clinical data |
| ☒ | ☐ Dual use research of concern |
| ☒ | ☐ Plants |

## Methods

| n/a | Involved in the study |
|---|---|
| ☒ | ☐ ChIP-seq |
| ☒ | ☐ Flow cytometry |
| ☒ | ☐ MRI-based neuroimaging |

## Plants

Seed stocks

*Report on the source of all seed stocks or other plant material used. If applicable, state the seed stock centre and catalogue number. If plant specimens were collected from the field, describe the collection location, date and sampling procedures.*

Novel plant genotypes

*Describe the methods by which all novel plant genotypes were produced. This includes those generated by transgenic approaches, gene editing, chemical/radiation-based mutagenesis and hybridization. For transgenic lines, describe the transformation method, the number of independent lines analyzed and the generation upon which experiments were performed. For gene-edited lines, describe the editor used, the endogenous sequence targeted for editing, the targeting guide RNA sequence (if applicable) and how the editor was applied.*

Authentication

*Describe any authentication procedures for each seed stock used or novel genotype generated. Describe any experiments used to assess the effect of a mutation and, where applicable, how potential secondary effects (e.g. second site T-DNA insertions, mosiacism, off-target gene editing) were examined.*
