# Supplementary material for: Stable cortical body maps before and after arm amputation
Source: Nat Neurosci. Author manuscript; Available in PMC 2025 Sep 30. (PMC12477700; doi:10.1038/s41593-025-02037-7)
Supplement: Supplementary file 2 [file NIHMS2109957-supplement-Supplementary_Table_1.pdf]

	P1	P2	P3
**Sex**	Female	Female	Female
**Age** (at first scan)	26	57	49
**Handedness at birth**	Left-handed	Right-handed	Right-handed
**Cause of amputation**	Arteriovenous vascular malformation (AVM)	Sarcoma tumour	Severell-Martorell syndrome led to multi-fractured arm with bones not healing
**Disability duration**	AVM progressed over a few years	Tumour slowly developing since 1995	Musculoskeletal issues since childhood
**Amputated limb**	Left upper limb	Right upper limb	Left upper limb
**Level of amputation**	Transhumeral	At elbow	Transhumeral
**Amputation surgery**	Combination of targeted muscle reinnervation and regenerative peripheral nerve interfaces, see Supplementary Figure 2.	Traditional: sharply transected the nerves and allowed to retract	Traditional: sharply transected the nerves and allowed to retract
**Phantom position and mobility**	Phantom hand positioned slightly above the elbow; only feels the hand, not the forearm; can move all phantom fingers (Figure 1B).	Phantom hand positioned upright towards chest; only feels the hand, not the forearm; can move all phantom fingers (Figure 1B).	Phantom hand positioned upright towards chest; mostly hand and fingers (little elbow); can move all phantom fingers (Figure 1B).
**When did phantom sensations occur**	Immediately after amputation	Immediately after amputation	Immediately after amputation
**Phantom limb sensation** (PLS) **intensity** (100 max) (3m, 6m, 1.5/5yrs respectively)	40, 60, 40	90, 100, 100	100, 90, NA
**PLS frequency** (3m, 6m, 1.5/5yrs)	3m: once a week; 6m: several times per month; 1.5yr: once or less per month	3m: all the time; 6m: all the time; 5yrs: all the time	3m: all the time; 6m: daily
**Chronic PLS** (100 max) (3m, 6m, 1.5/5yrs)	13.3, 15, 8	90, 100, 100	100, 45, NA
**Limb pain intensity** (Pre, 3m, 6m, 1.5/5yrs)	90, 20, 0, 0	80, 50, 70, 70	50, 80, 70, NA
**Limb pain frequency** (Pre, 3m, 6m, 1.5/5yrs)	Pre: all the time; 3m: several times per month; 6m: once or less per month; 1.5yr: once or less per month	Pre: all the time; 3m: daily; 6m: daily; 5yrs: all the time	Pre: daily; 3m: daily; 6m: once a week
**Chronic limb pain** (Pre, 3m, 6m, 1.5/5yrs)	90, 5, 0, 0	80, 25, 35, 70	25, 40, 23.3, NA
**Transient** (on the day) **limb pain** (Pre, 3m, 6m, 1.5/5yrs; 100 max) (Pre, 3m, 6m, 1.5/5yrs)	50, 30, 0, 0	80, 45, 50, 70	50, 40, 20, NA
**Pain Detect Score (% max possible score)** (Pre, 3m, 6m, 1.5/5yrs)	51%, 34%, 14%, 40%	68%, NA, 42%, 45%	65%, 65%, 65%, NA
**Pain Detect Pain Course**	- Persistent pain with pain attacks (Same pre and 3m)<br>- Persistent pain with slight fluctuations (6m, 1.5yrs)	- Persistent pain with pain attacks (Same pre and 6m)<br>- Persistent pain with slight fluctuations (5yrs)	- Pain attacks with pain between them (pre)<br>- Persistent pain with pain attacks (3m)<br>- Pain attacks without pain between them (6m)
**Upper Extremity Functional Index** (Pre, 3m, 6m, 1.5/5yrs) 100% = no impairment	47%, 23%, 36%, 57%	30%, NA, 11%, 28%	0%, 39% 69%, NA
**Prosthesis Type**	None	None (fitted with a cosmetic prosthetic)	Cosmetic prosthesis
**Prosthesis Use**	None	None. Briefly used in the first 6 months post-amputation (2 days a week, ~2 hours a day)	6m: 2 days a week, 8 hours a day
